# Two decades of the synthesis of mono- and bis-aminomercapto[1,2,4]triazoles

**DOI:** 10.1039/d0ra04208k

**Published:** 2020-07-01

**Authors:** Sayed M. Riyadh, Sobhi M. Gomha

**Affiliations:** Department of Chemistry, Faculty of Science, Taibah University Al-Madinah Al-Munawarah 30002 Saudi Arabia; Department of Chemistry, Faculty of Science, Cairo University Giza 12613 Egypt s.m.gomha@gmail.com; Department of Chemistry, Faculty of Science, Islamic University in Al-Madinah Al-Munawarah 42351 Saudi Arabia

## Abstract

4-Amino-5-mercapto[1,2,4]triazole and its 3-substituted derivatives have proven to be of biological interest and provide access to a new class of biologically active heterocyclic compounds for biomedical applications. This study will be helpful for scientific researchers interested in the chemistry of bifunctional versatile compounds as it provides a collection of all the methods for the preparation of 3-substituted-4-amino-5-mercapto[1,2,4]triazoles with aliphatic, aromatic, and heterocyclic moieties during the period from 2000 to mid-2020.

## Abstract 2

4-Amino-5-mercapto[1,2,4]triazole and its 3-substituted derivatives have proven to be of biological interest and provide access to a new class of biologically active heterocyclic compounds for biomedical applications.

## Graphical abstract

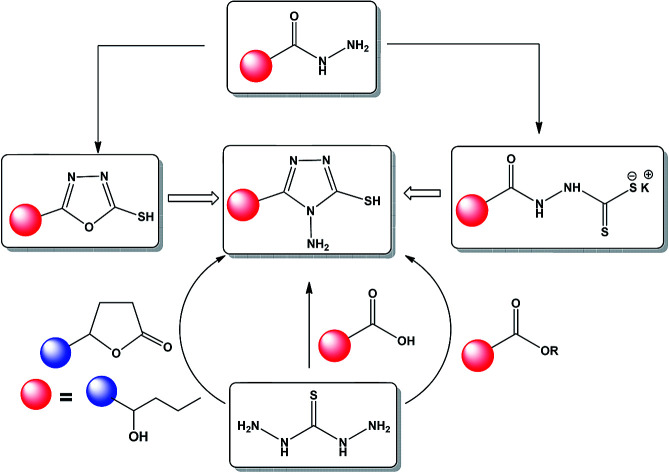

## 1. Introduction

**Chart 1:**
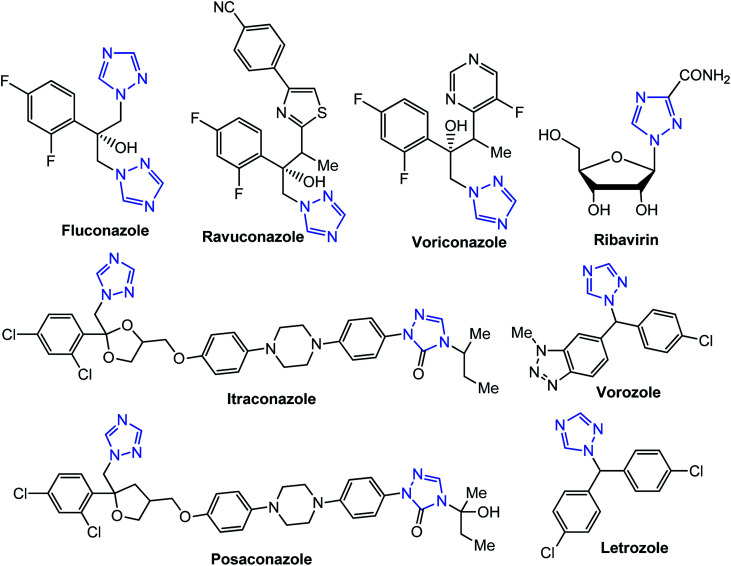
Examples of [1,2,4]triazole bearing drugs.

[1,2,4]Triazoles and their fused heterocyclic derivatives have occupied a unique position as novel biologically active agents with remarkably diverse pharmacological properties such as antimicrobial, antifungal, anticancer, anticonvulsant, antiviral, anti-inflammatory, anti-HIV, and anti-mycobacterial activities.^1–8^ A large number of ring systems containing [1,2,4]triazoles have been incorporated into a wide variety of therapeutically interesting drug candidates such as fluconazole, ravuconazole, itraconazole, voriconazole, posaconazole, vorozole, letrozole, ribavirin, triazolam, alprazolam, etizolam, furacylin, hexaconazole, triadimefon, myclobutanil, rizatriptan, propiconazole, and fluotrimazole (Chart 1).^9^ Moreover, the synthesis of bis-heterocyclic compounds containing triazole rings has attracted attention due to the diverse applications of these compounds in numerous pharmacological and biological fields.^10–13^

**Chart 2:**
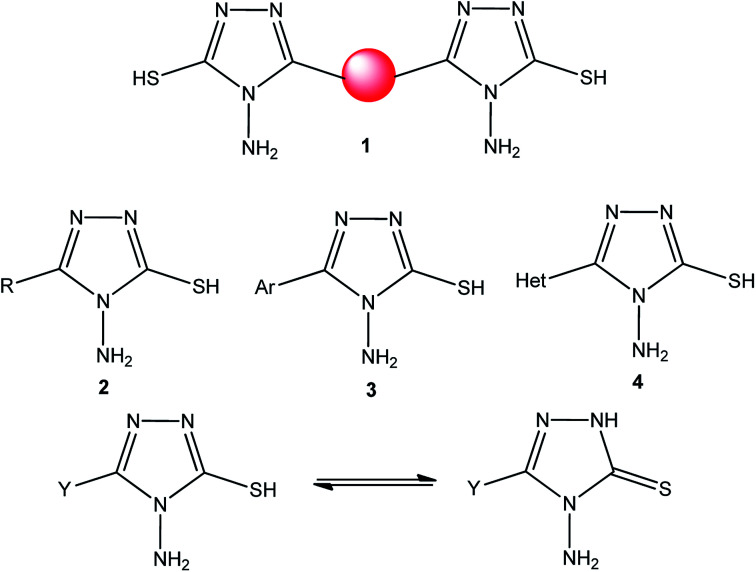
Structures of bis-[4-amino-5-mercapto[1,2,4]triazoles] (1) and 3-substituted-4-amino-5-mercapto[1,2,4]triazoles (2–4).

Bis-[4-amino-5-mercapto[1,2,4]triazoles] (1) and 3-substituted-4-amino-5-mercapto[1,2,4] triazoles (2–4) (Chart 2) contain both amino and mercapto groups as ready-made nucleophilic centers for the synthesis of condensed heterocyclic rings. The introduction of these groups in different nuclei enhances their biological activities. Accordingly, the objective of the present review is to highlight the synthetic methods used to obtain 3-substituted-4-amino-5-mercapto[1,2,4]triazoles and bis-[4-amino-5-mercapto[1,2,4]triazoles] from 2000 until mid-2020.

## 2. Synthetic routes using thiocarbohydrazide as the precursor

### 2.1. Reactions with carboxylic acids

**Scheme 1:**
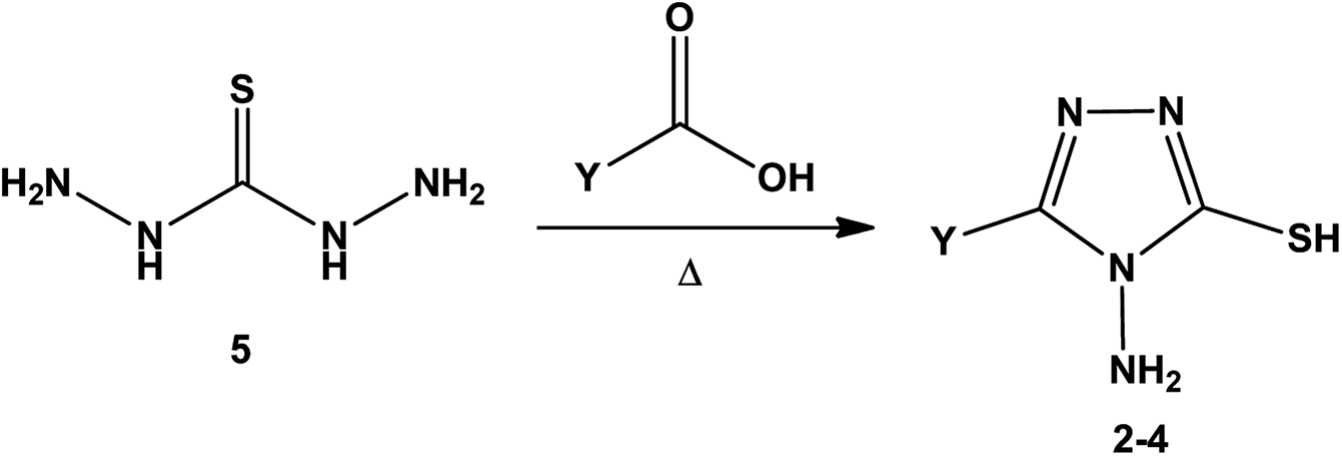
Synthesis of triazoles 2–4.

3-Substituted-4-amino-5-mercapto[1,2,4]triazoles 2–4 were prepared from the treatment of thiocarbohydrazide (5) with carboxylic acids (Scheme 1) (Table 1).

**Table 1:** Derivatives of 3-substituted-4-amino-5-mercapto[1,2,4]triazoles

Y	Ref.
H, –CH_3_, –C_2_H_5_	14
–CH_3_	15 and 16
–CH_3_, –CF_3_	17
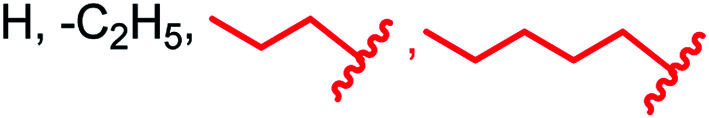	18
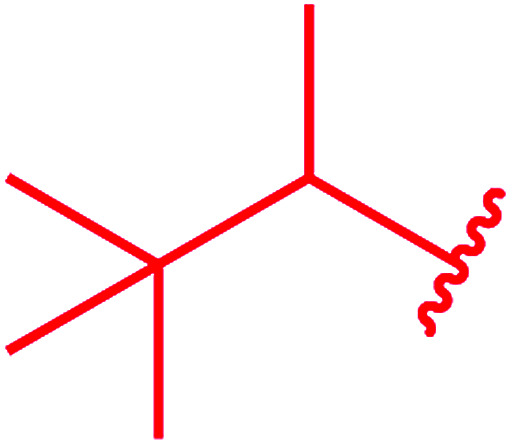	19
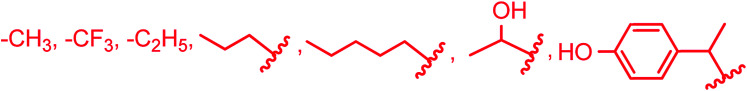	20
Substituted phenyl	21–23
Ar–CH_2_–CH_2_– & cyclohexyl–CH_2_–CH_2_–	24
Ar–O–CH_2_– & Ar–NH–CH_2_– & Ar–S–CH_2_– & Ar–SO_2_NH–CH_2_– & Ar–CONH–CH_2_– & Ar–CH(CH_3_)– & triazole–CH_2_–	25
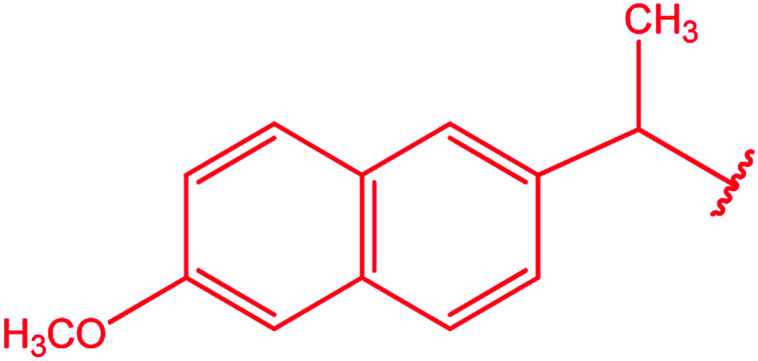	26
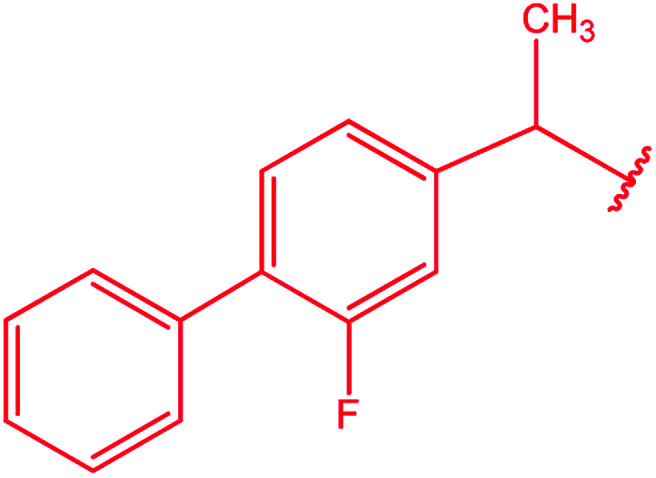	26
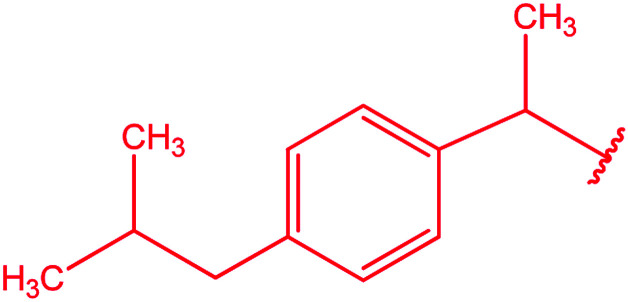	26 and 27
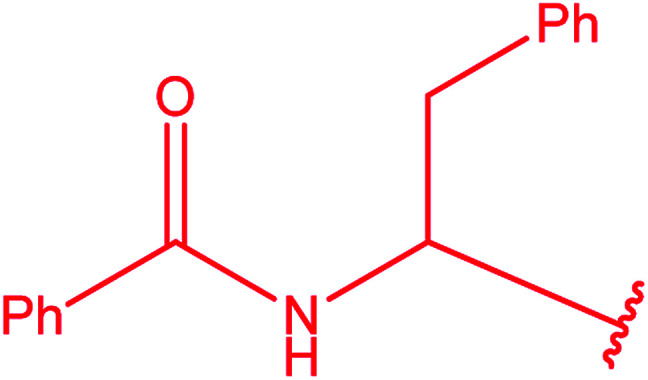	28
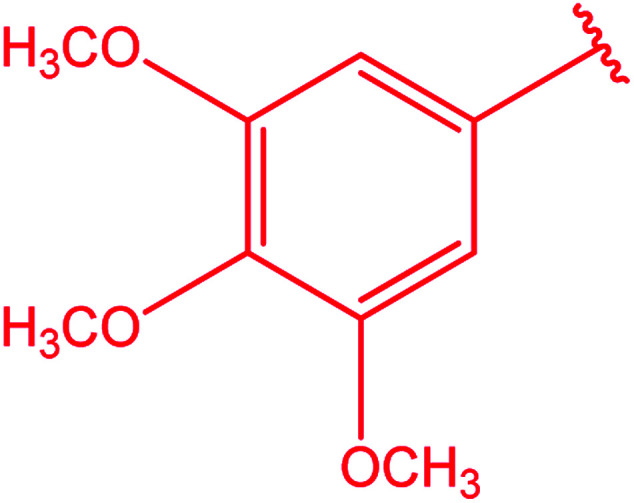	29
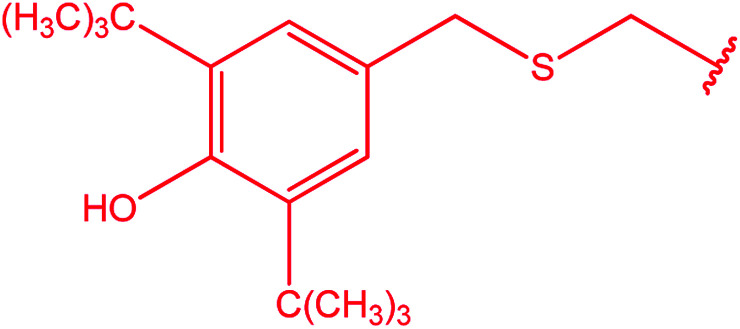	30
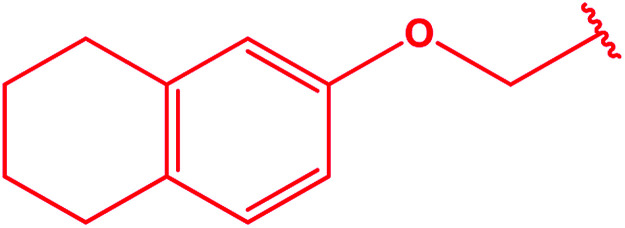	31
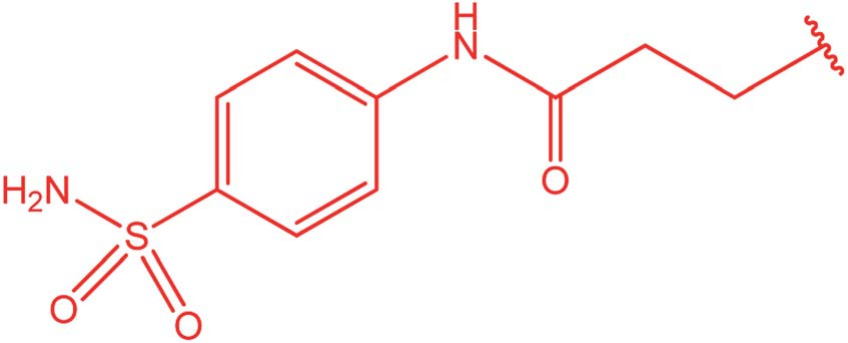	32
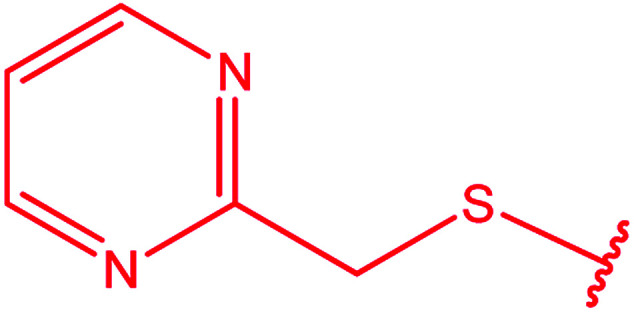	33
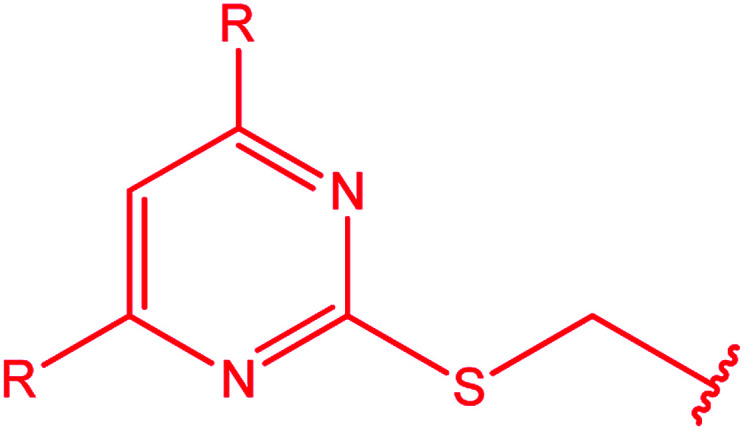	34
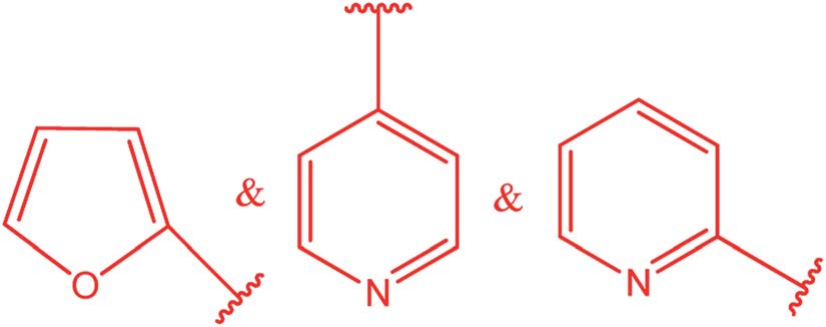	35
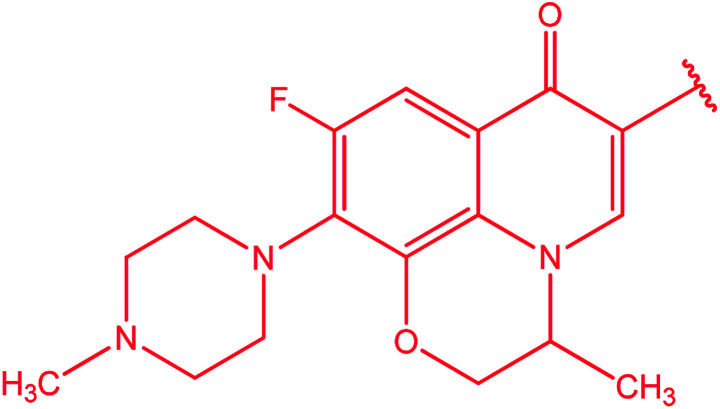	36
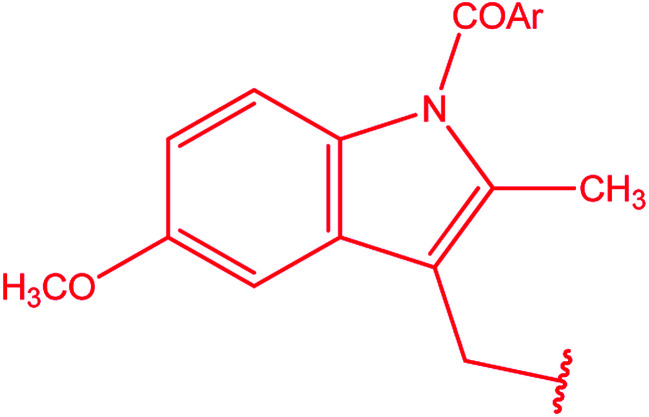	37
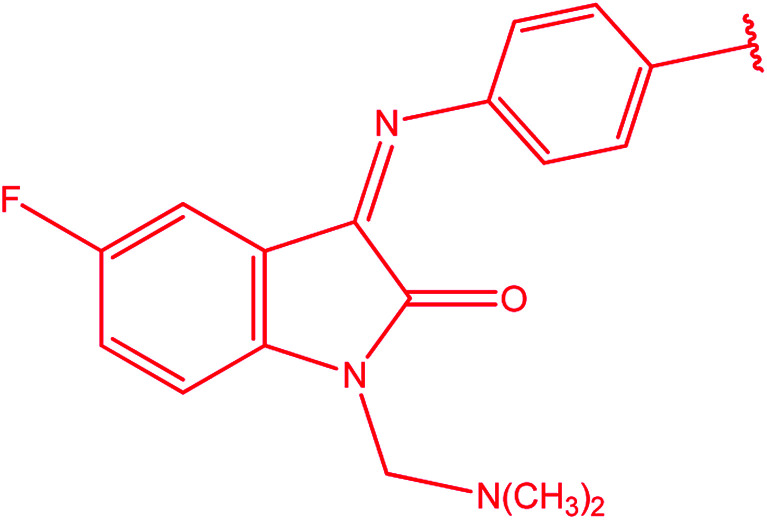	38
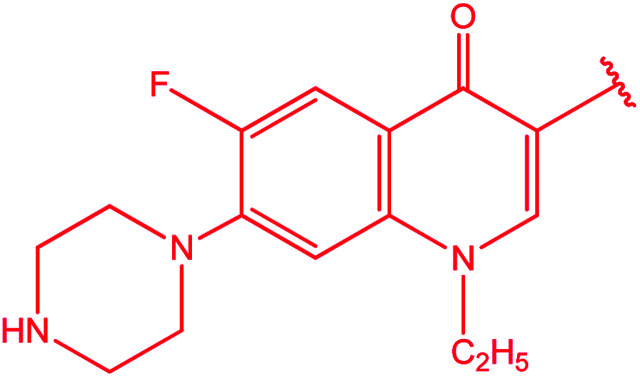	39
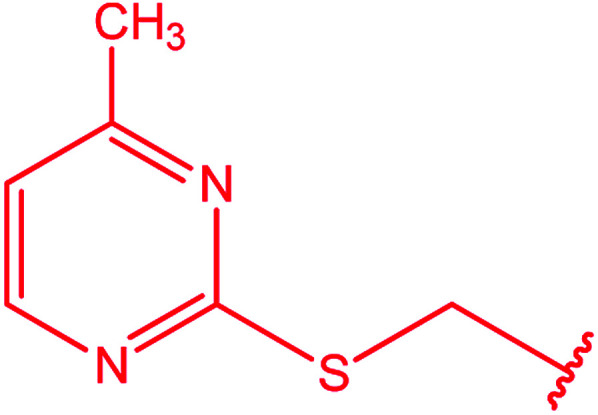	40
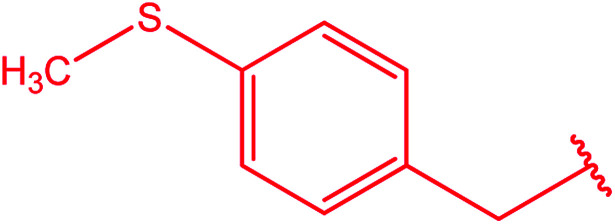	9

**Scheme 2:**
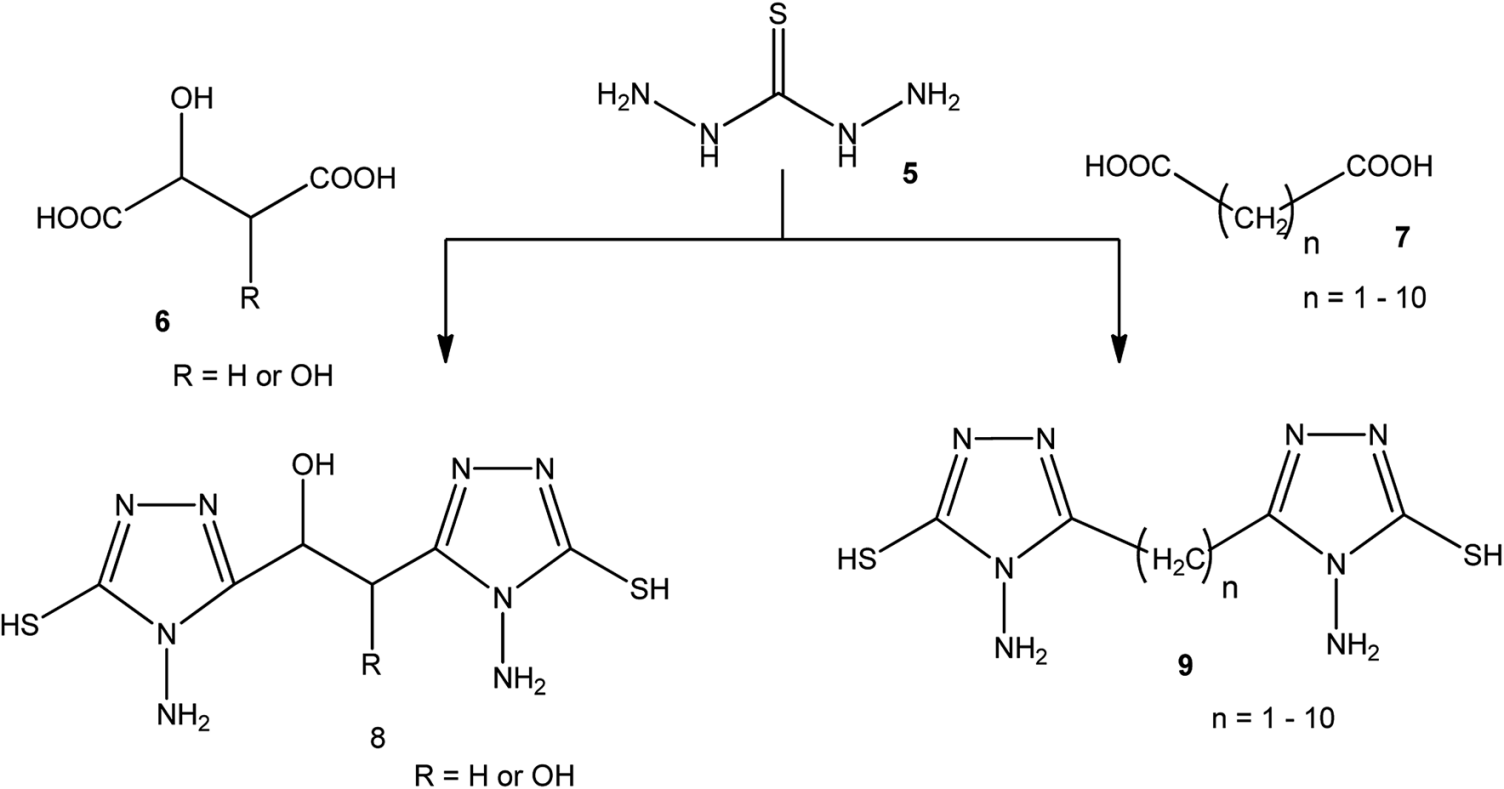
Synthesis of bis-triazoles 8 and 9.

A series of dicarboxylic acids such as tartaric, malic,^41–43^ succinic,^44^ glutaric,^45^ and others^46^ were treated with thiocarbohydrazide (5) to afford the respective series of bis-(4-amino-5-mercapto[1,2,4]triazoles) 8, 9 (Scheme 2).

**Scheme 3:**
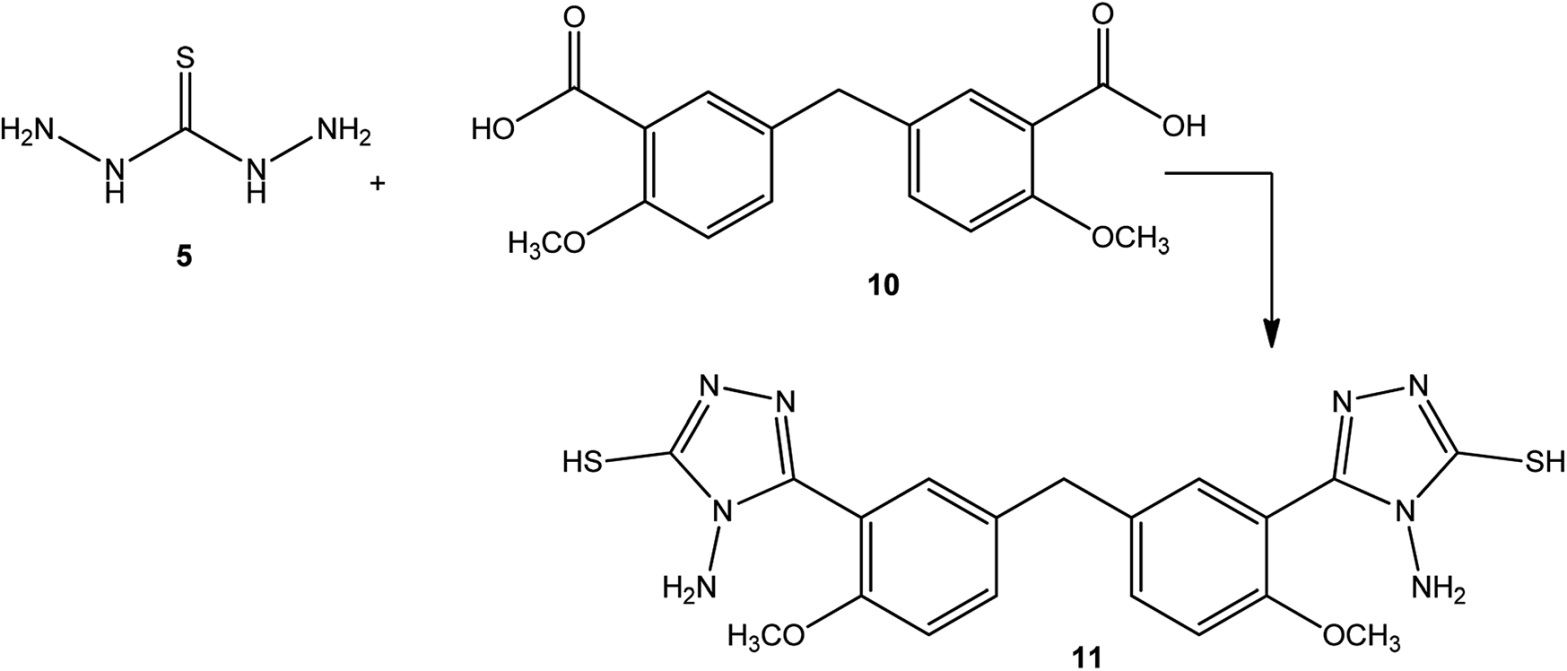
Synthesis of bis-triazole 11.

Similarly, a condensation reaction between 5-(3-formyl-4-methoxybenzyl)-2-methoxybenzoic acid (10) and thiocarbohydrazide (5) at the melt temperature afforded bis[4-methoxy-3-[4-amino-5-sulfanyl-4*H*-1,2,4-triazol-3-yl]phenyl]methane (11) (Scheme 3).^47^

### 2.2. Reactions with esters

**Scheme 4:**
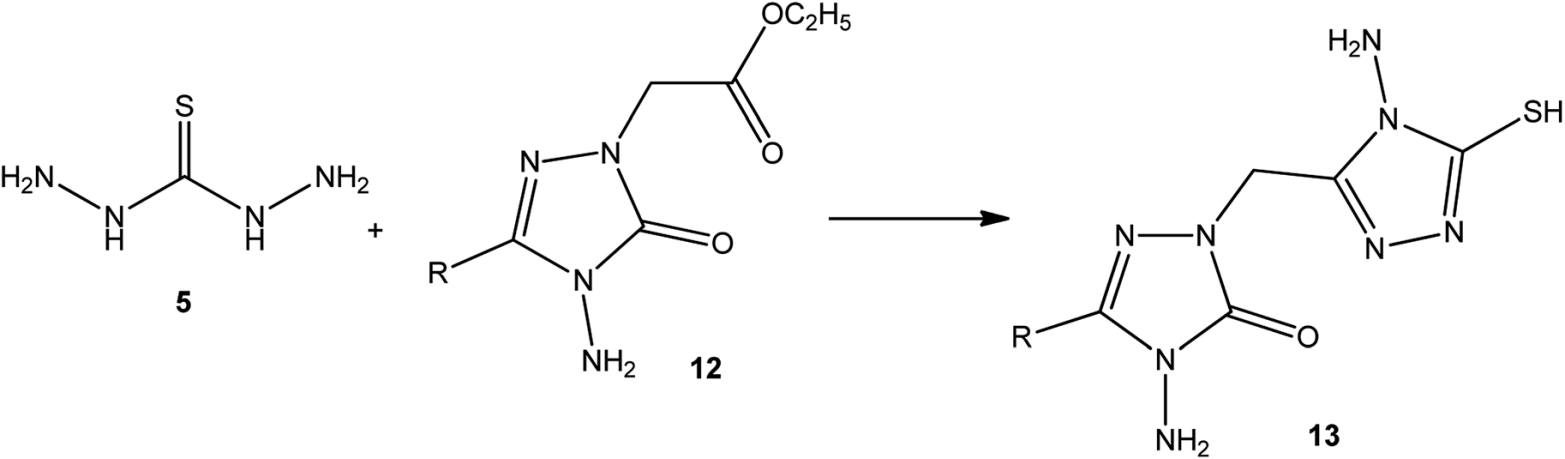
Synthesis of triazoles 13.

In addition, Demirbas *et al.*^48^ reported the treatment of ethyl(3-alkyl-4-amino-5-oxo-4,5-dihydro-1*H*-1,2,4-triazol-1-yl) acetates (12) with thiocarbohydrazide (5), which furnished 5-alkyl-4-amino-2-[(4-amino-5-mercapto-4*H*-1,2,4-triazol-3-yl)methyl]-2,4-dihydro-3*H*-1,2,4-triazol-3-ones (13) (Scheme 4).

**Scheme 5:**
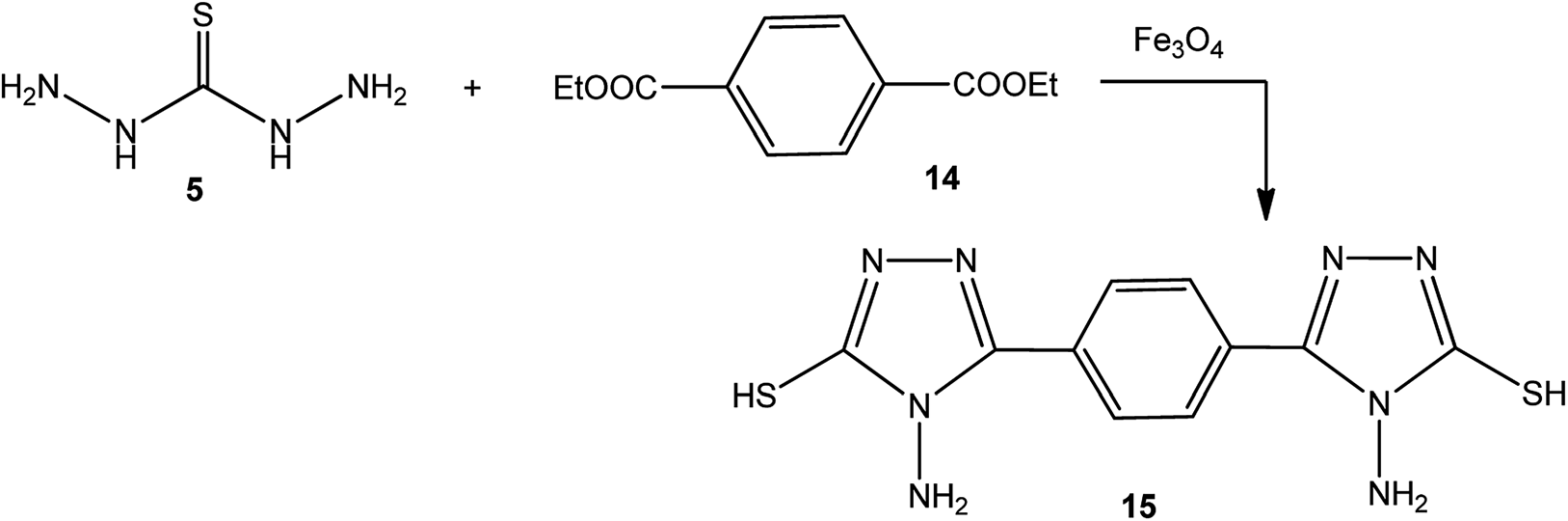
Synthesis of bis-triazole 15.

Moreover, refluxing thiocarbohydrazide (5) with diethyl terephthalate 14 using magnetic iron oxide (Fe_3_O_4_) nanoparticles as an eco-friendly catalyst yielded the respective 3,3′-(1,4-phenylene)bis(4-amino-1*H*-1,2,4-triazole-5(4*H*)-thione) (15) (Scheme 5).^49^

### 2.3. Reactions with lactones

**Scheme 6:**
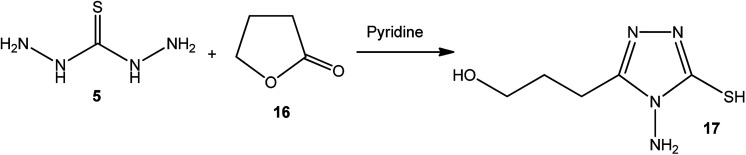
Synthesis of triazole 17.

4-Amino-3-(3-hydroxypropyl)-5-mercapto[1,2,4]triazole (17) was prepared *via* the treatment of thiocarbohydrazide (5) with lactone 16, as reported by Zhang *et al.*^50^ [Scheme 6].

**Scheme 7:**
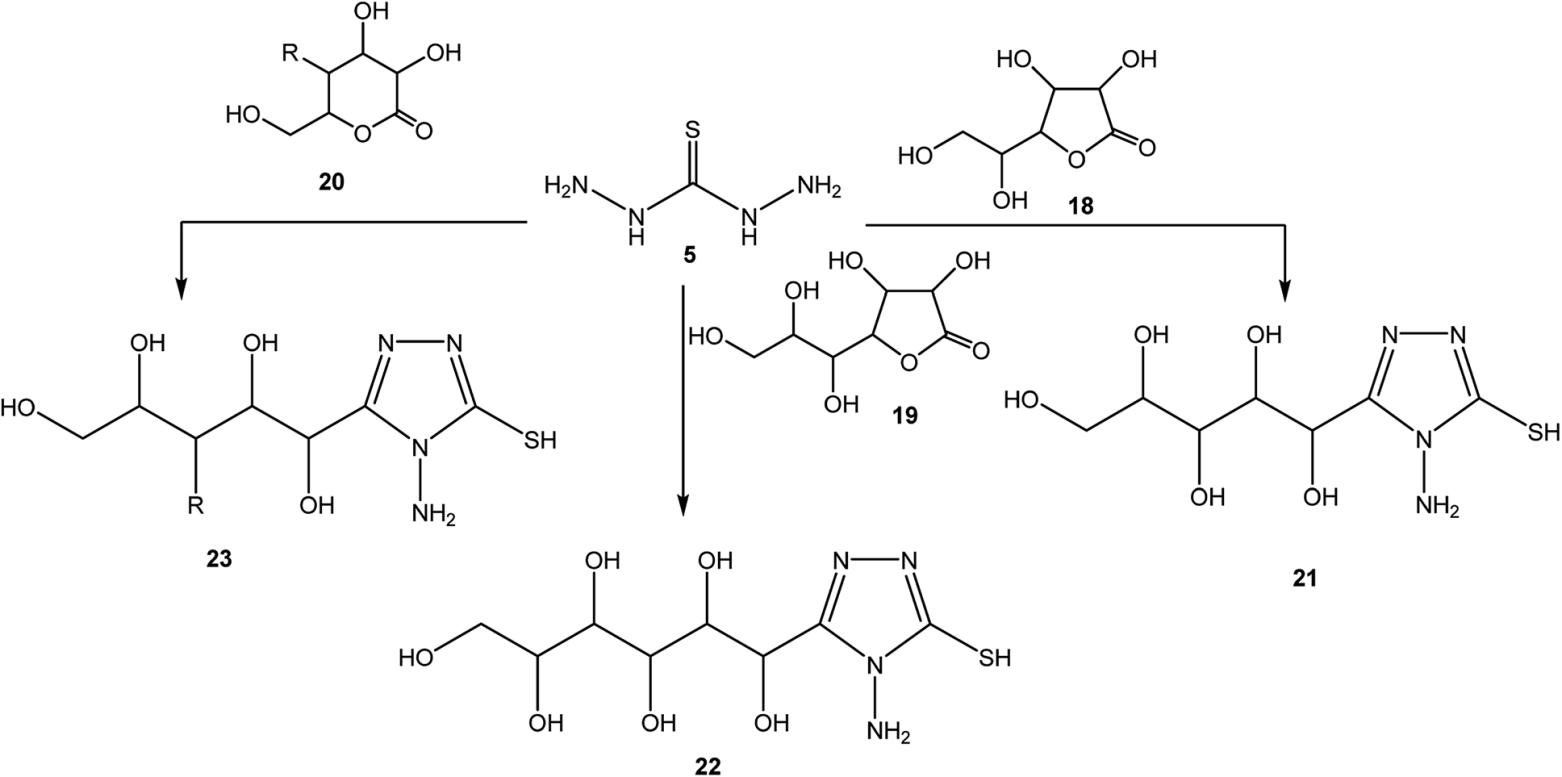
Synthesis of triazoles 21–23.

The synthetic routes for the preparation of 4-amino-3-(d-galactopentitol-1-yl)-5-mercapto[1,2,4]triazole (21),^51^ 4-amino-3-(d-glucoheptonic-hexitol-1-yl)-1*H*-[1,2,4]triazole-5-thione (22),^52^ and 3-(d-alditol-1-yl)-4-amino-5-mercapto-[1,2,4]triazole (23)^53^ were reported through reactions of thiocarbohydrazide (5) with d(−)galactono-1,4-lactone (18), d-glucoheptonic-γ-lactone (19), and d-galactono-1,5-lactones (20), respectively (Scheme 7).

## 3. Use of potassium acyldithiocarbazates with hydrazine hydrate

**Scheme 8:**
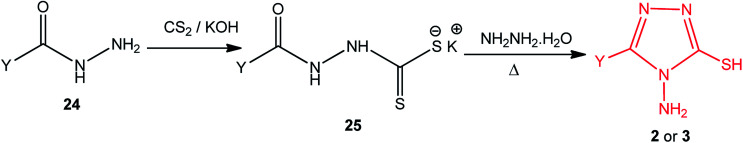
Synthesis of triazoles 2 and 3.

Potassium acyldithiocarbazates 25 is usually prepared by a reaction between the corresponding acid hydrazides 24 and carbon disulfide in an ethanolic potassium hydroxide solution. This method was extensively used in the synthesis of numerous derivatives of 4-amino-5-mercapto[1,2,4]triazoles 2 (3) upon treatment with hydrazine hydrate (Scheme 8) (Table 2).

**Table 2:** Derivatives of 3-substituted-4-amino-5-mercapto[1,2,4]triazoles

Y	Ref.
–CH_3_, –C_2_H_5_, –C_3_H_7_	54
CH_3_–(CH_2_)_13_–CH_2_–	55
CH_3_–(CH_2_)_15_–CH(SO_3_Na)–	56
C_6_H_5_–	57–60
3-ClC_6_H_4_–	8
4-CH_3_OC_6_H_4_–	61
2-HOC_6_H_4_–	62
2-CH_3_C_6_H_4_– & 2-CH_3_-4-ClC_6_H_3_–	63
C_6_H_5_– & 2-HOC_6_H_4_–	64
2-C_2_H_5_OC_6_H_4_–	65
3-Br-4-CH_3_OC_6_H_3_–	66
2-HOC_6_H_4_– & 4-HOC_6_H_4_– & 4-C_2_H_5_OC_6_H_4_– & 2-HO-5-ClC_6_H_3_– & 4-HOC_6_H_4_–CH_2_– & 4-C_2_H_5_OC_6_H_4_–CH_2_–	67
2-FC_6_H_4_–CH_2_– & 2-BrC_6_H_4_–CH_2_– & 4-HOC_6_H_4_–CH_2_– & 2-CH_3_OC_6_H_4_–CH_2_– & 4-NO_2_C_6_H_4_–CH_2_–	68
C_6_H_5_– & 2-ClC_6_H_4_– & 2-NO_2_C_6_H_4_– & 2-HOC_6_H_4_– & 2-furyl	69
C_6_H_5_– & 4-ClC_6_H_4_– & 4-BrC_6_H_4_– & 4-CH_3_OC_6_H_4_– & 2-naphthyl–CH_2_–	70
2-HOC_6_H_4_– & 4-HOC_6_H_4_– & 2-NH_2_C_6_H_4_– & 4-NH_2_C_6_H_4_– & 3,4,5-(HO)_3_C_6_H_2_–	71
C_6_H_5_–CH_2_–CH_2_–	72
2-(CH_3_)_2_NC_6_H_4_– & 4-CH_3_NHC_6_H_4_– & 1-naphthyl–CH_2_–	73
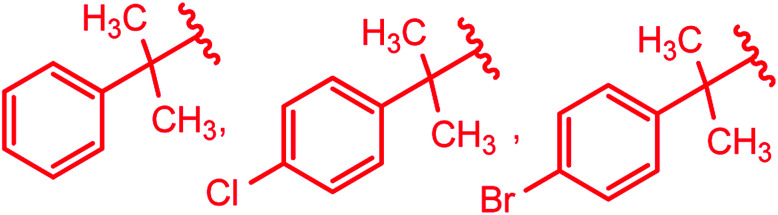	74
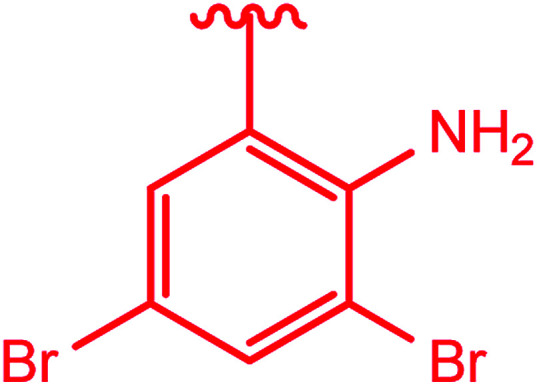	75
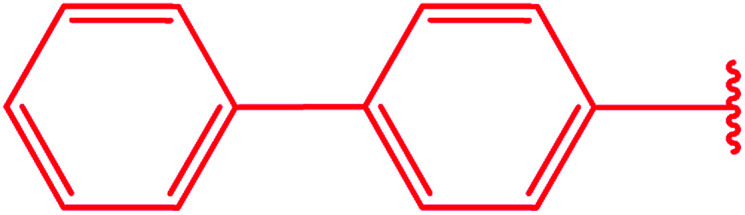	76
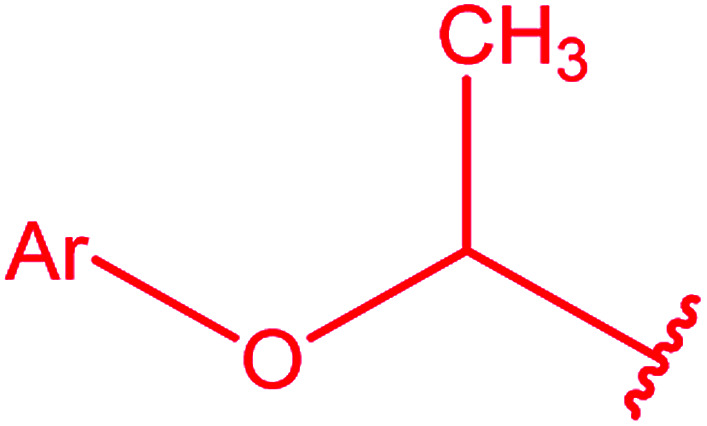	77
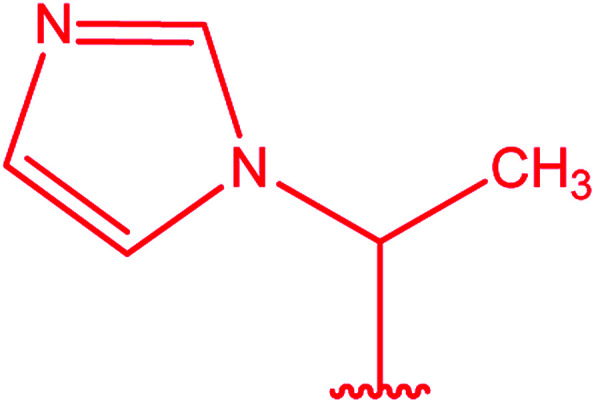	77
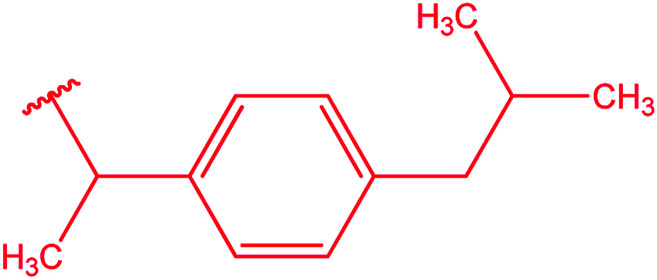	78 and 79
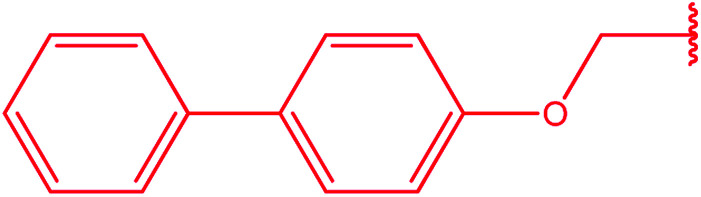	79
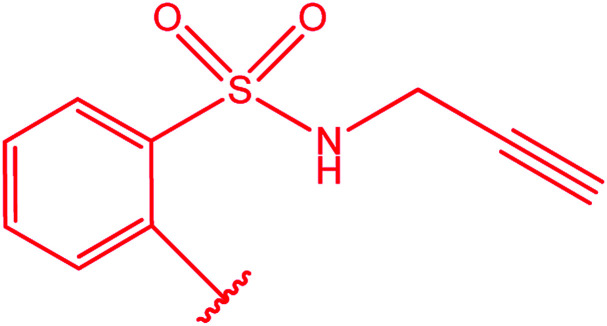	80
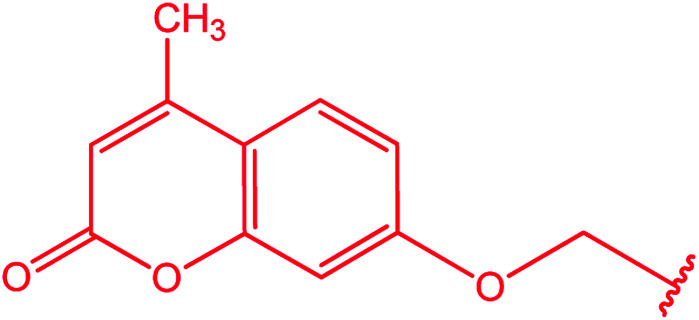	81
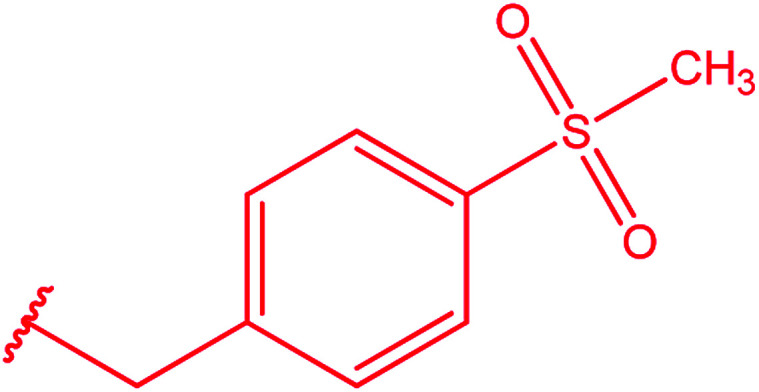	82
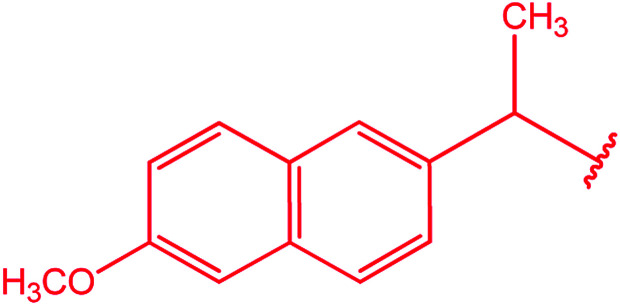	83
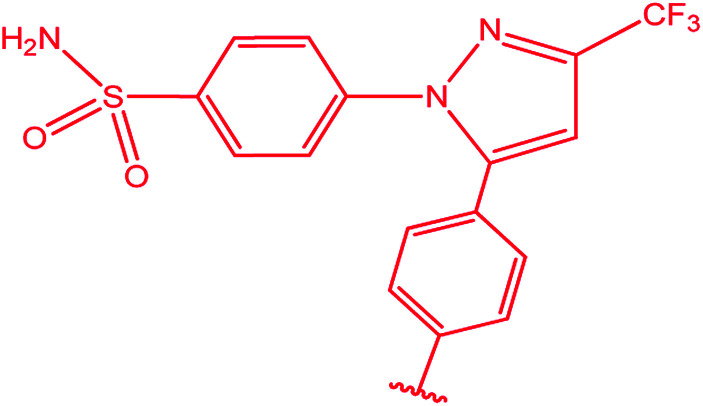	84
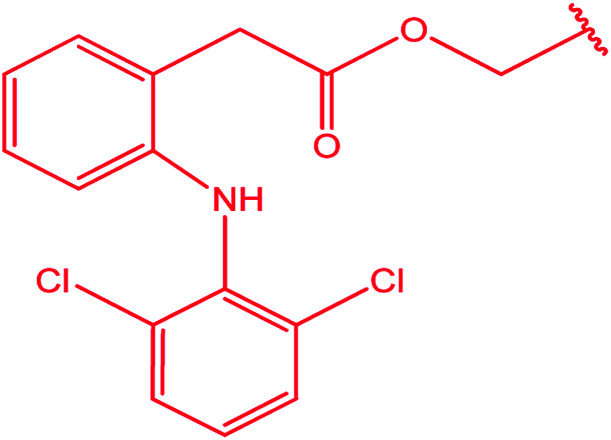	85
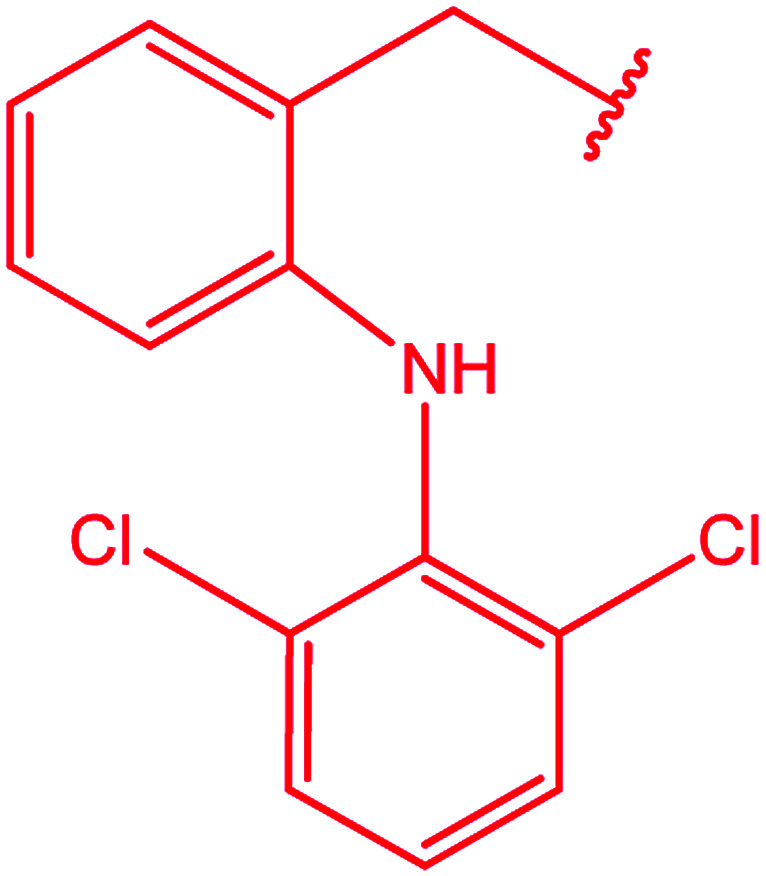	85 and 86
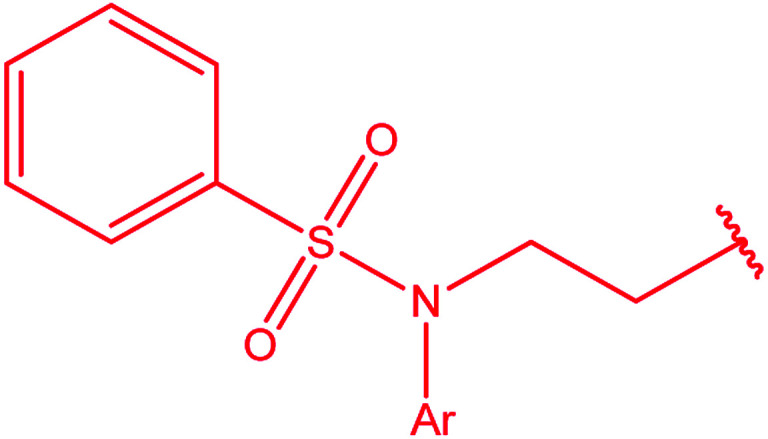	87
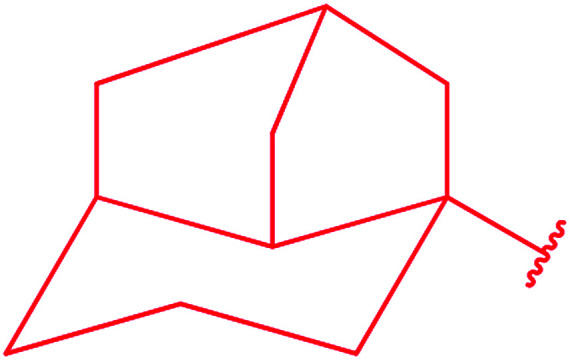	88

**Scheme 9:**
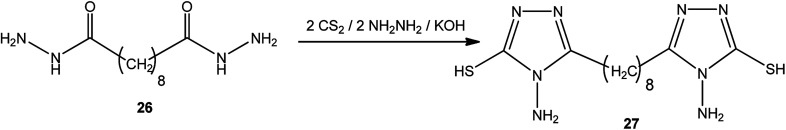
Synthesis of bis-triazole 27.

1,8-Bis-(3-mercapto-4-amino-[1,2,4]-triazol-5-yl)-octane (27) was achieved *via* the reaction of sebacic acid dihydrazide (26) with carbon disulfide and hydrazine hydrate in a molar ratio of 1 : 2 : 2 in the presence of potassium hydroxide^89^ (Scheme 9).

**Scheme 10:**
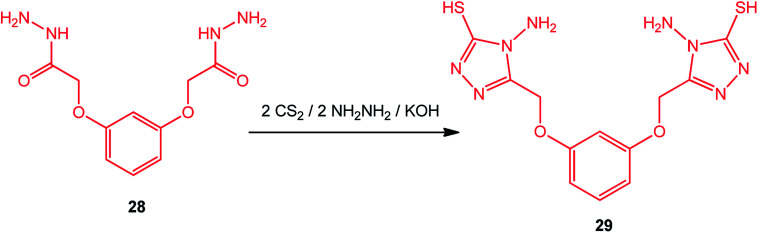
Synthesis of bis-triazole 29.

Bis-(3-mercapto-4-amino-[1,2,4]-triazole) with an aromatic moiety was prepared under similar conditions by Zhao *et al.*^90^ Thus, the reaction of 2,2′-[1,3-phenylenebis(oxy)]bis-acetic hydrazide (28) with CS_2_/NH_2_NH_2_ afforded 2,2′-[1,3-phenylenebis(oxymethylene)]bis-(4-amino-3-mercapto-[1,2,4]triazole) (29) (Scheme 10).

**Scheme 11:**
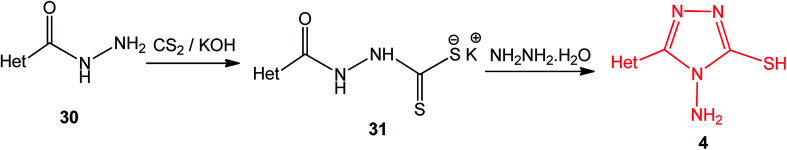
Synthesis of triazoles 4.

3-Heteroaryl-4-amino-5-mercapto[1,2,4]triazoles (4) were synthesized by the treatment of the corresponding dithiocarbazate 31 with hydrazine hydrate (Scheme 11) (Table 3).

**Table 3:** Derivatives of 3-substituted-4-amino-5-mercapto[1,2,4]triazoles

Het	Ref.
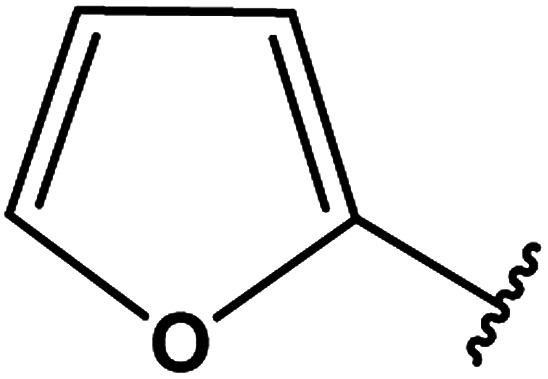	91
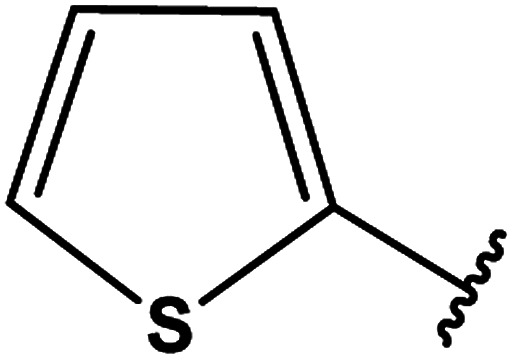	92
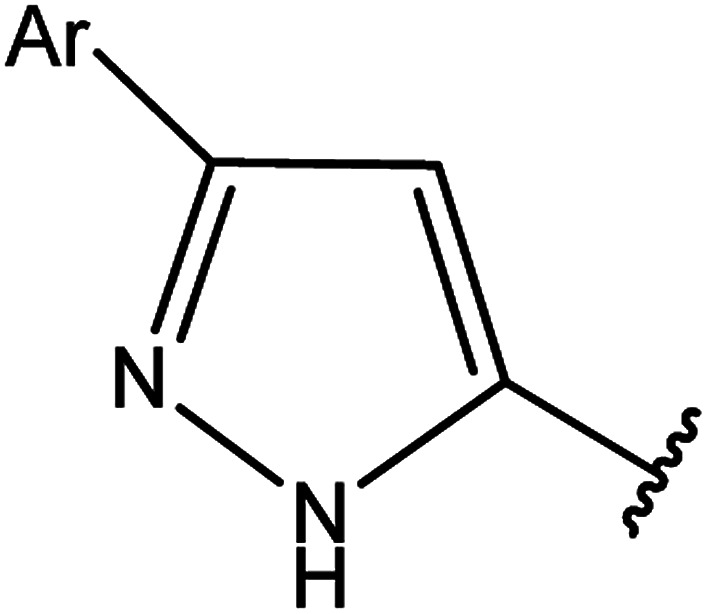	93
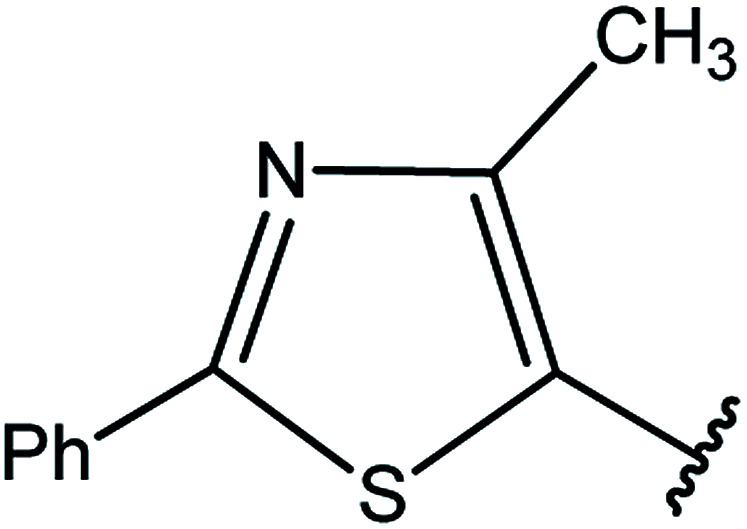	94
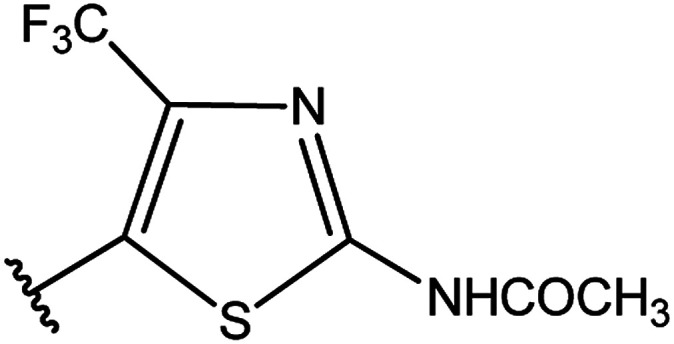	95
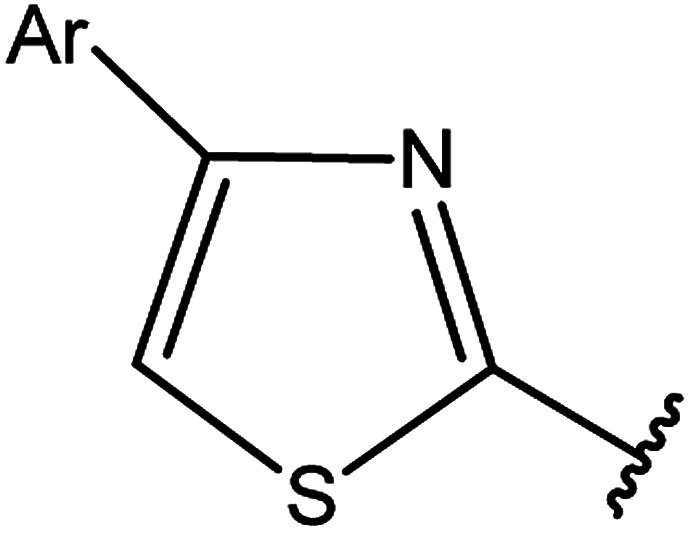	96
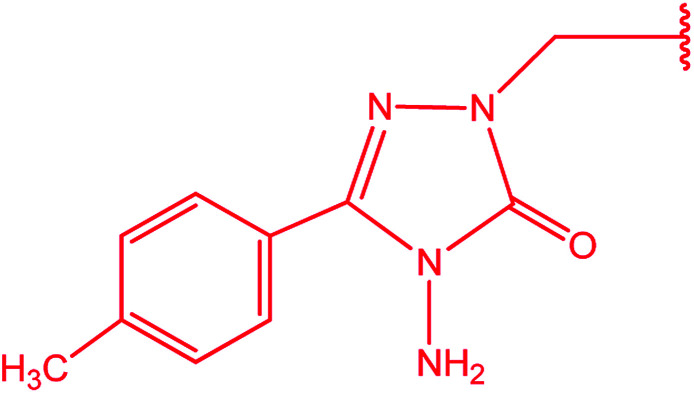	97
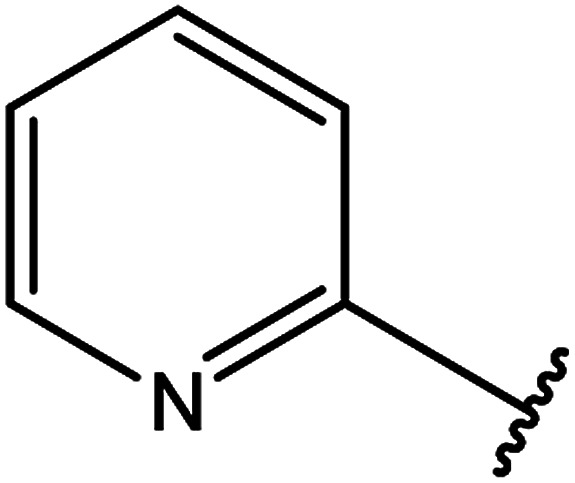	98
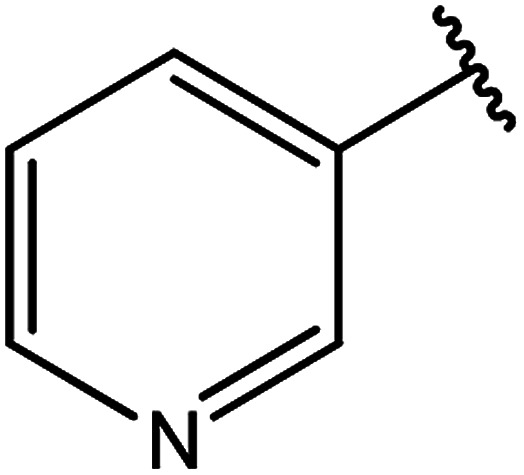	99
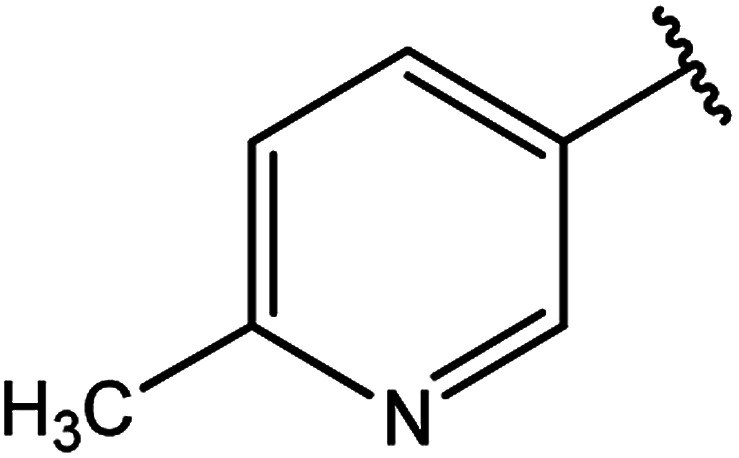	20
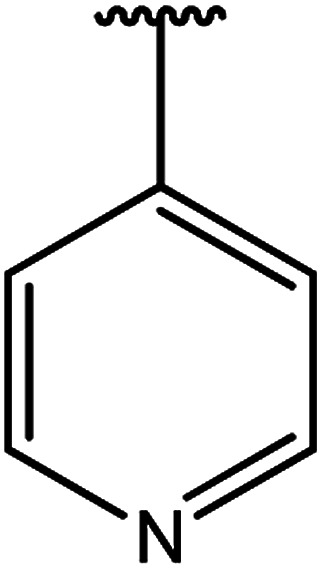	100–106
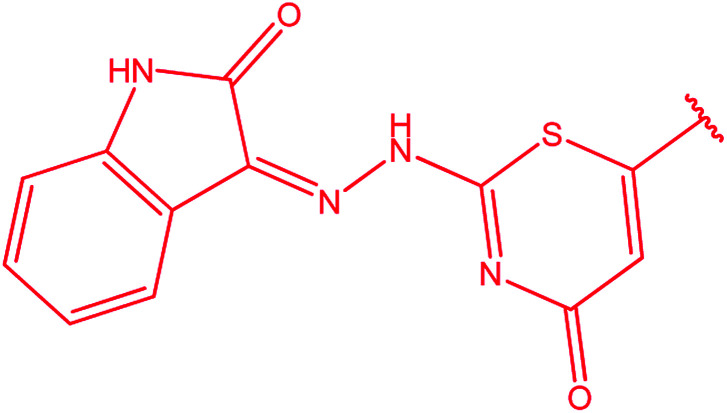	107
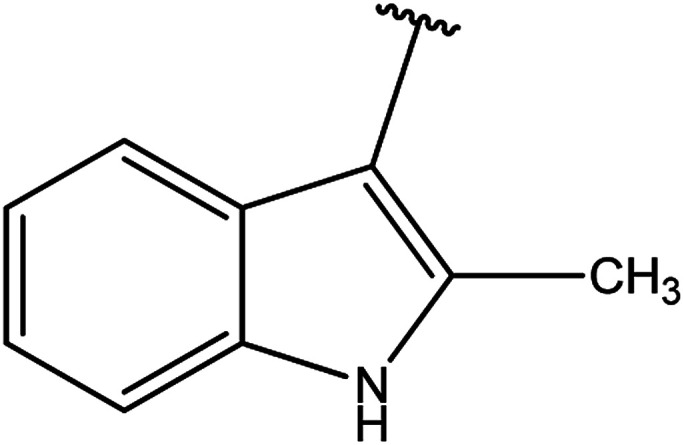	108
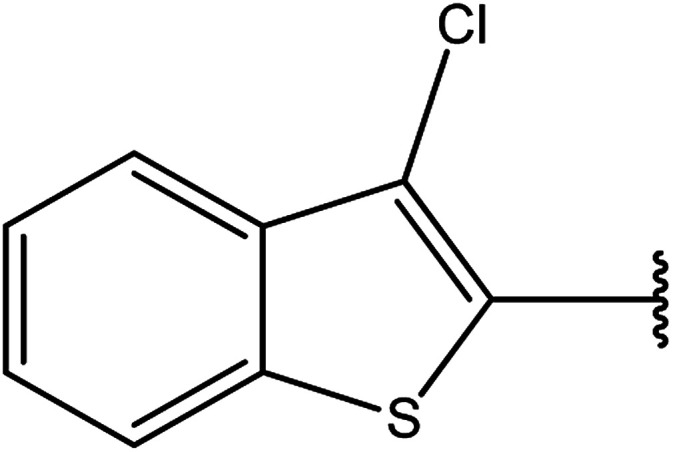	109
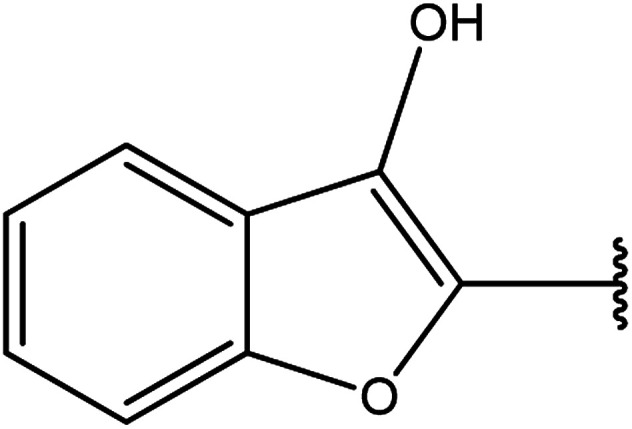	110
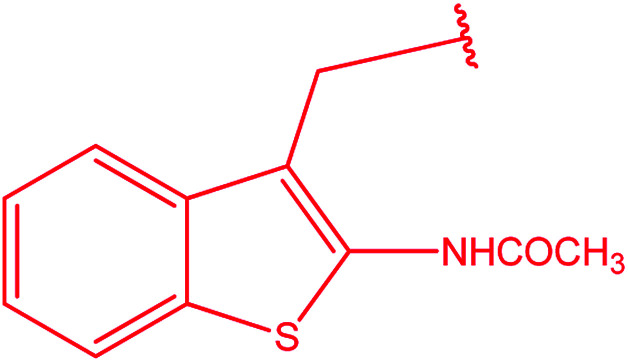	111
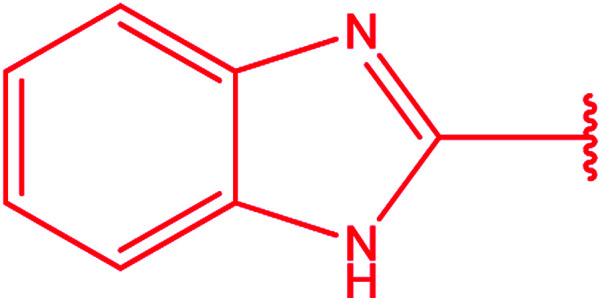	112
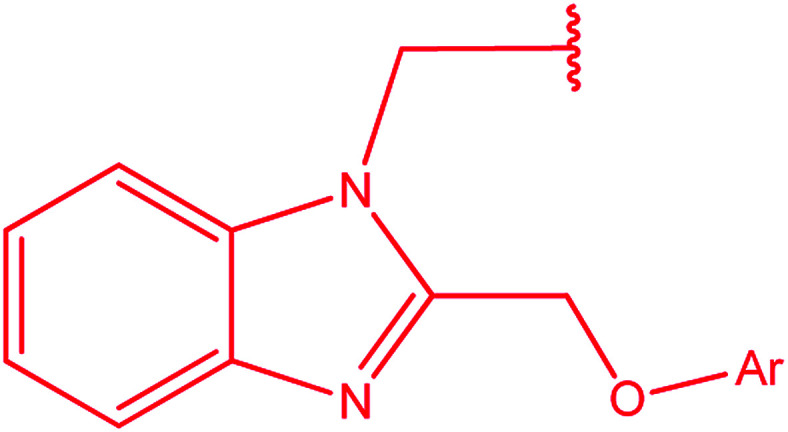	113
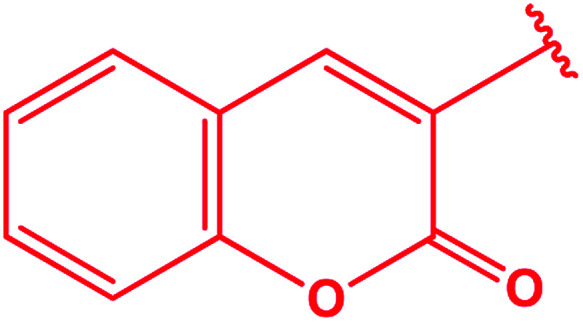	114
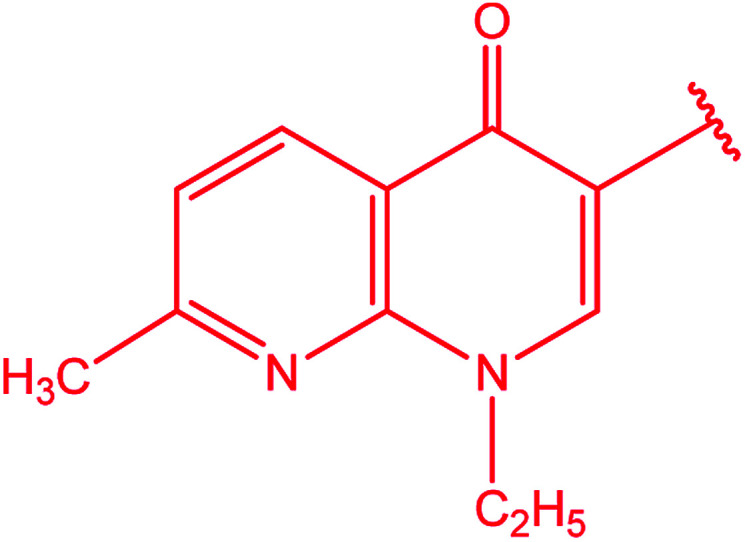	115
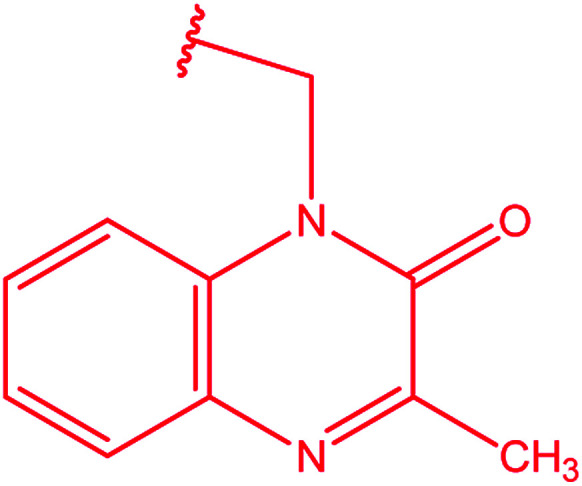	116
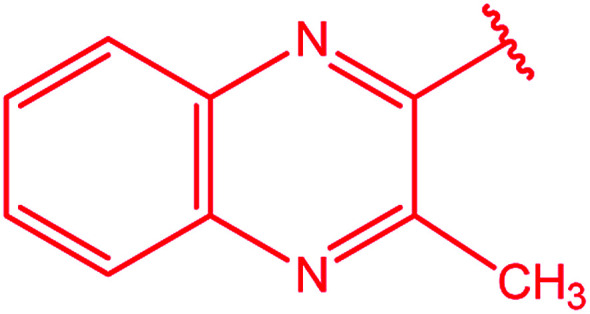	117
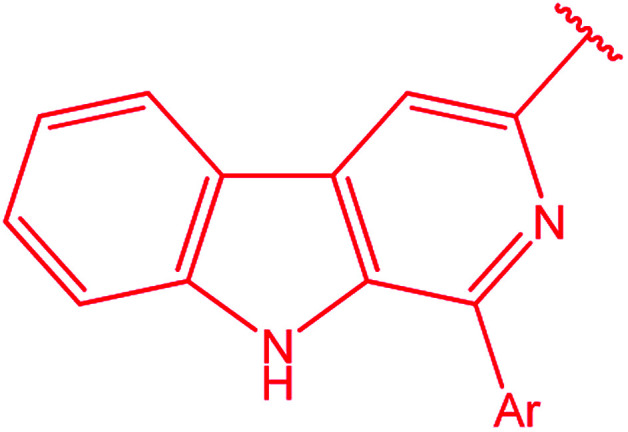	118
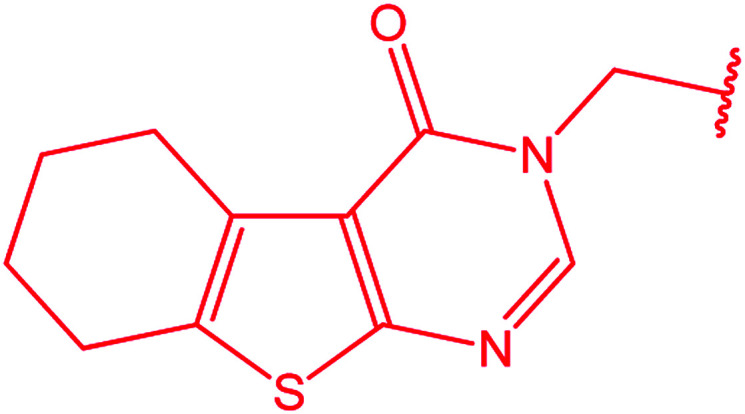	119
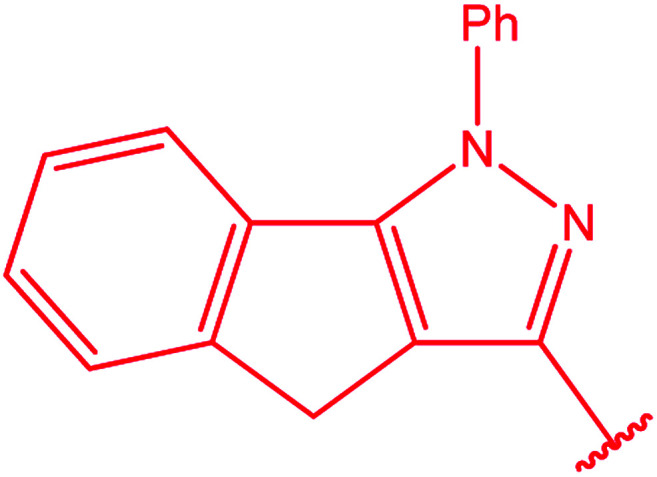	120

**Scheme 12:**
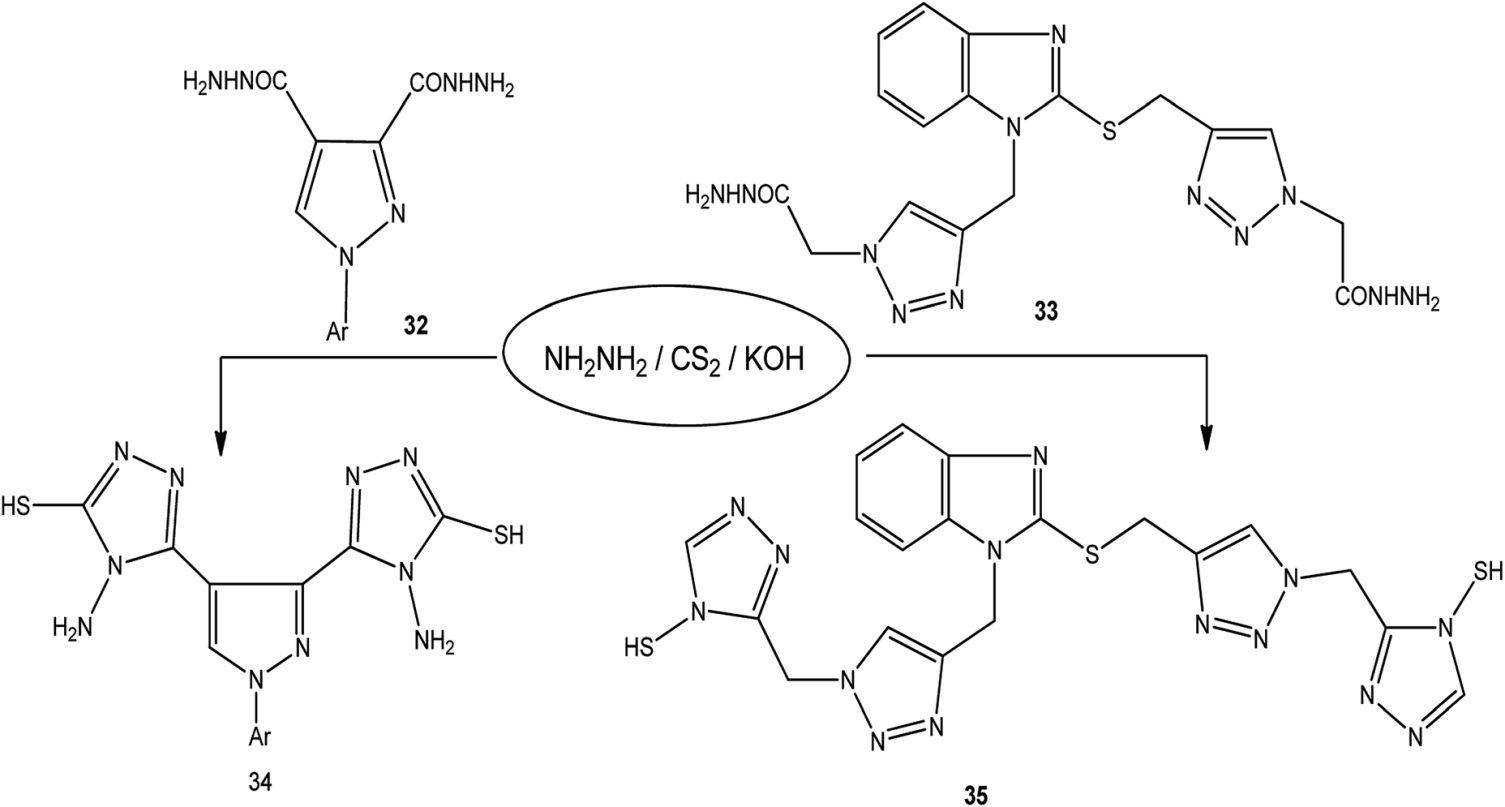
Synthesis of bis-triazoles 34 and 35.

The treatment of dicarbohydrazides 32 (ref. 121) and 33 (ref. 122) with CS_2_/NH_2_NH_2_ in the presence of KOH proceeded smoothly to afford the respective bis-triazoles 34 and 35 (Scheme 12).

**Scheme 13:**
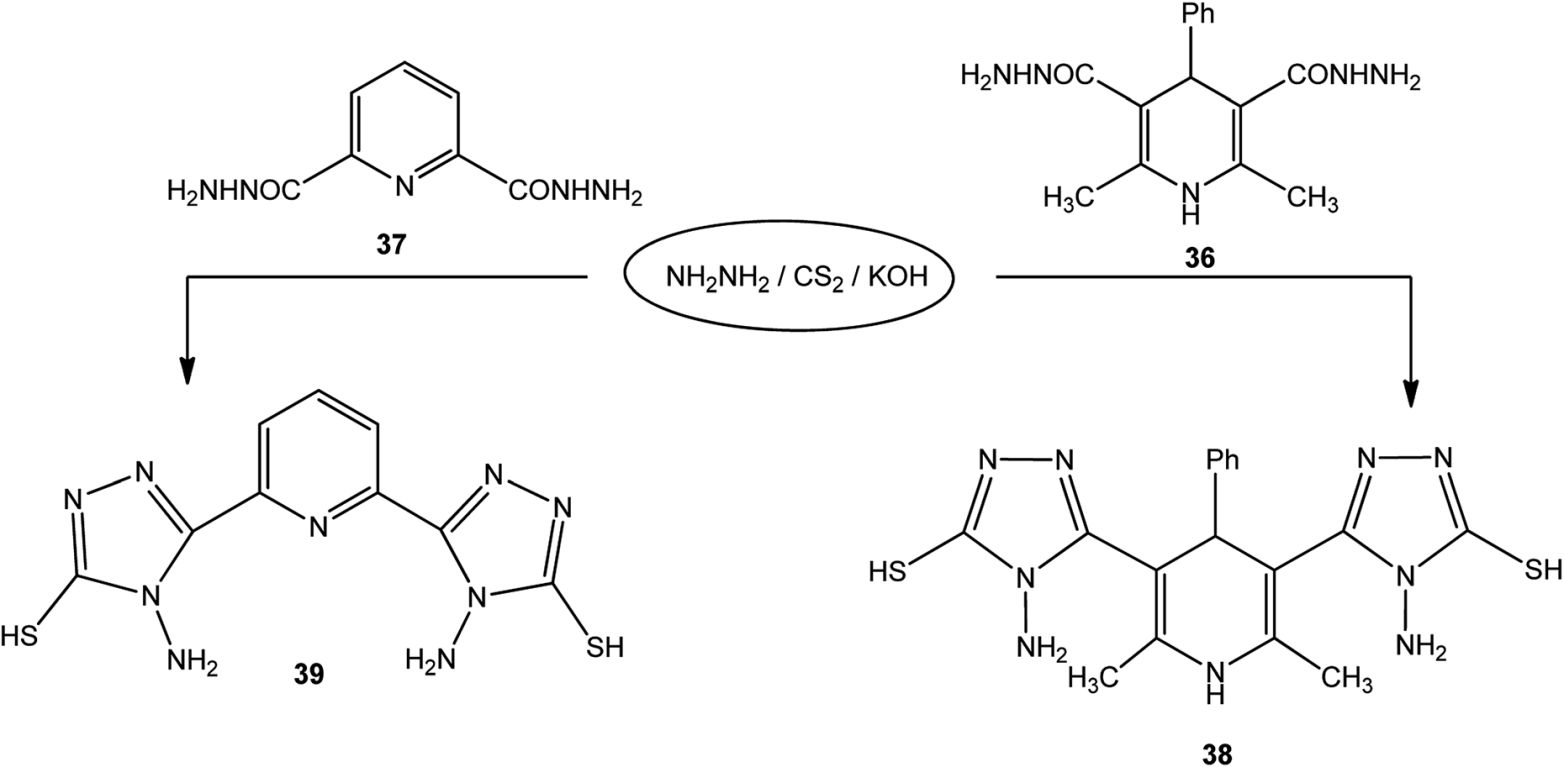
Synthesis of bis-triazoles 38 and 39.

In addition, pyridine dicarbohydrazide derivatives 36 (ref. 123) and 37 (ref. 124 and 125) were reacted with the above reagents under similar conditions to give 38 and 39, respectively (Scheme 13).

**Scheme 14:**
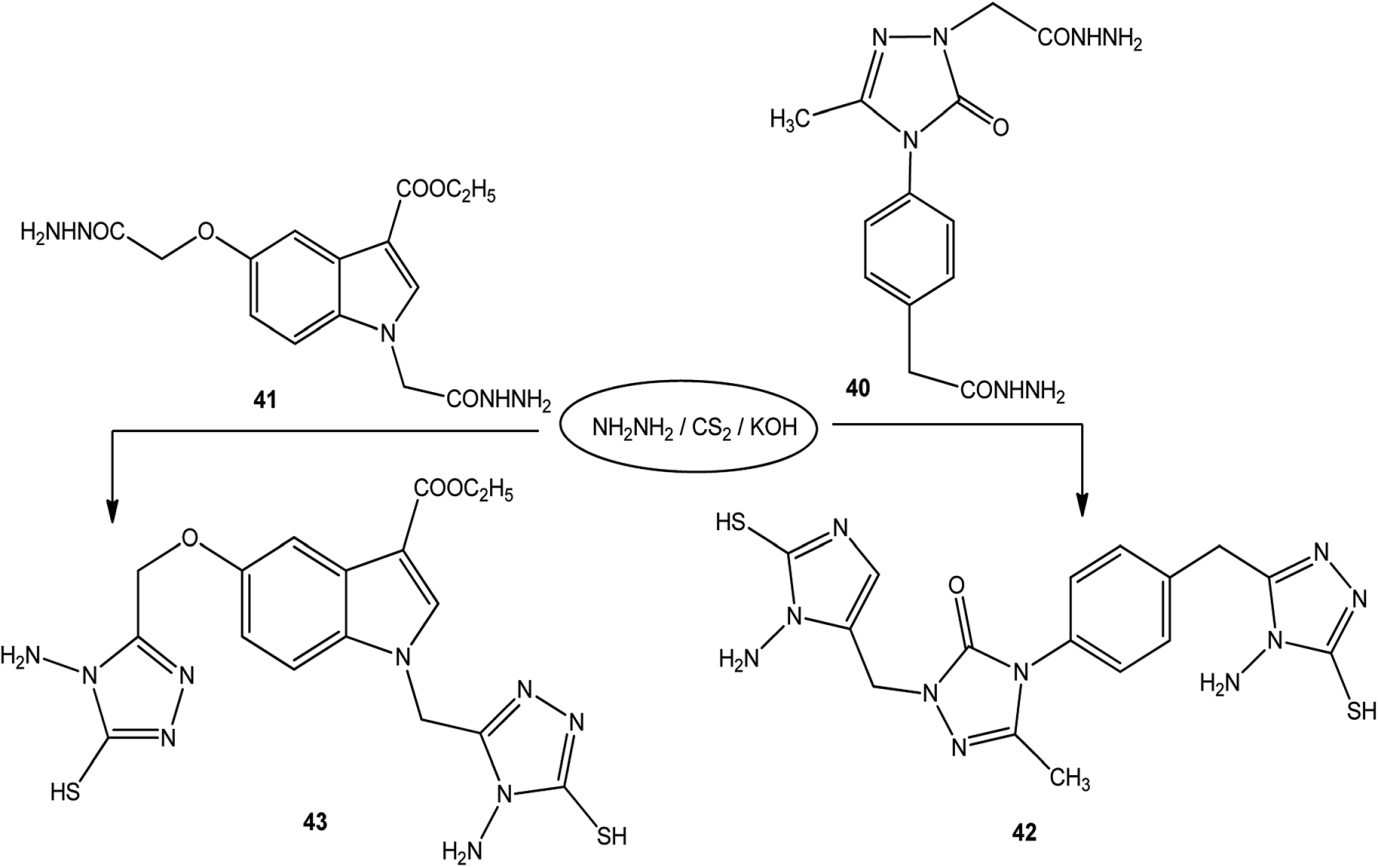
Synthesis of bis-triazoles 42 and 43.

Moreover, the reactions of dicarbohydrazide of triazole 40 (ref. 126) or indole derivatives 41 (ref. 127) with the same reagents in an alkaline solution furnished 42 or 43, respectively (Scheme 14).

## 4. Synthesis of 5-mercapto[1,3,4]oxadiazoles with hydrazine hydrate *via* ring transformation reactions

**Scheme 15:**
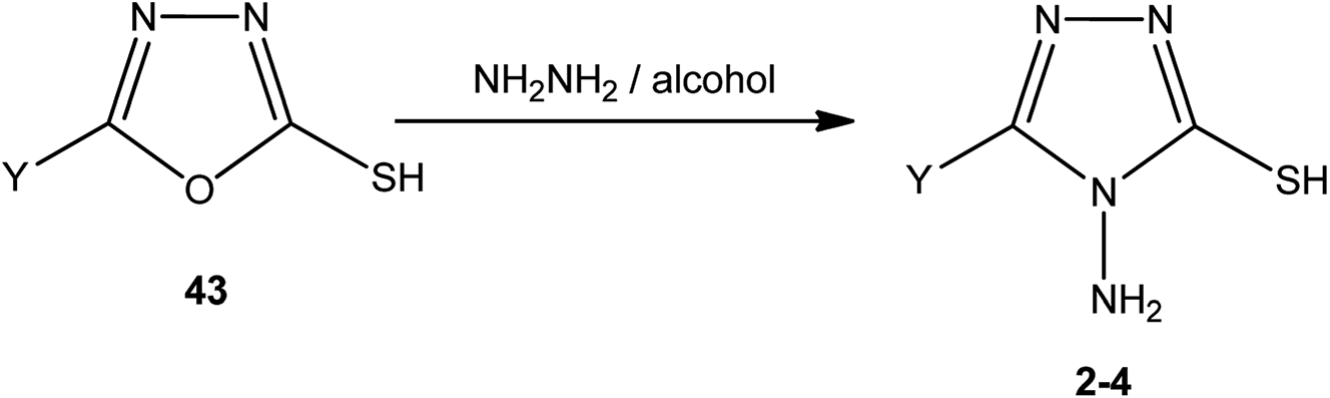
Synthesis of triazoles 2–4.

An alcoholic solution of hydrazine hydrate achieves the ring transformation of 3-substituted-5-mercapto[1,3,4]oxadiazoles (43) to 3-substituted-4-amino-5-mercapto[1,3,4]triazoles 2–4 (Scheme 15) (Table 4).

**Table 4:** Derivatives of 3-substituted-4-amino-5-mercapto[1,2,4]triazoles

Y	Ref.
CH_3_–CH_2_–CH_2_– & CH_3_–(CH_2_)_4_–CH_2_– & CH_3_–(CH_2_)_5_–CH_2_– & CH_3_–(CH_2_)_6_–CH_2_–	128
C_6_H_5_– & 4-NO_2_C_6_H_4_– & 3-NO_2_C_6_H_4_– & 3-NO_2_-4-ClC_6_H_3_– & 2-NH_2_-5-ClC_6_H_3_– & 4-CH_3_OC_6_H_4_– & 3,4,5-(OCH_3_)_3_C_6_H_2_– & C_6_H_5_CH_2_– & 1-naphthyl	128
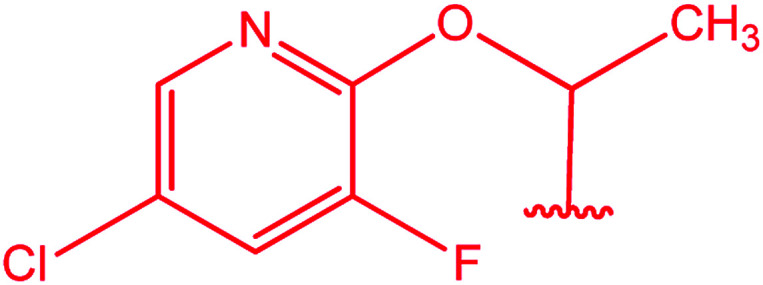	129
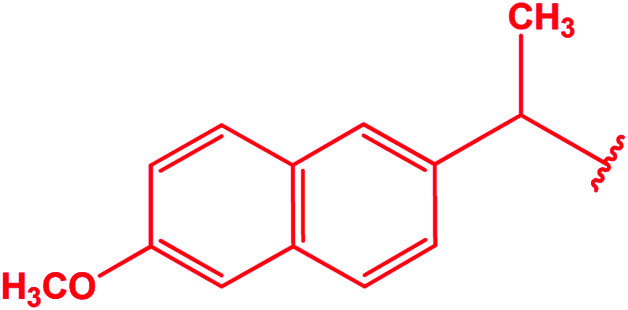	130
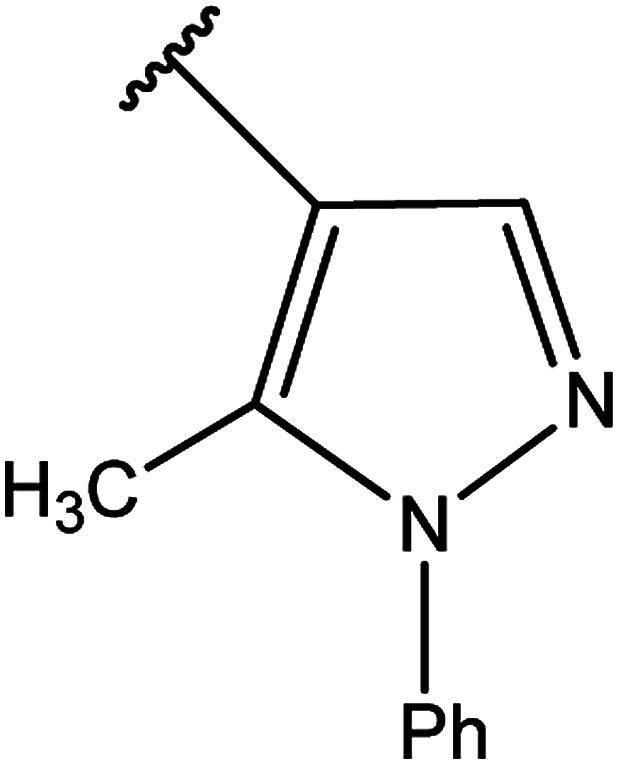	131
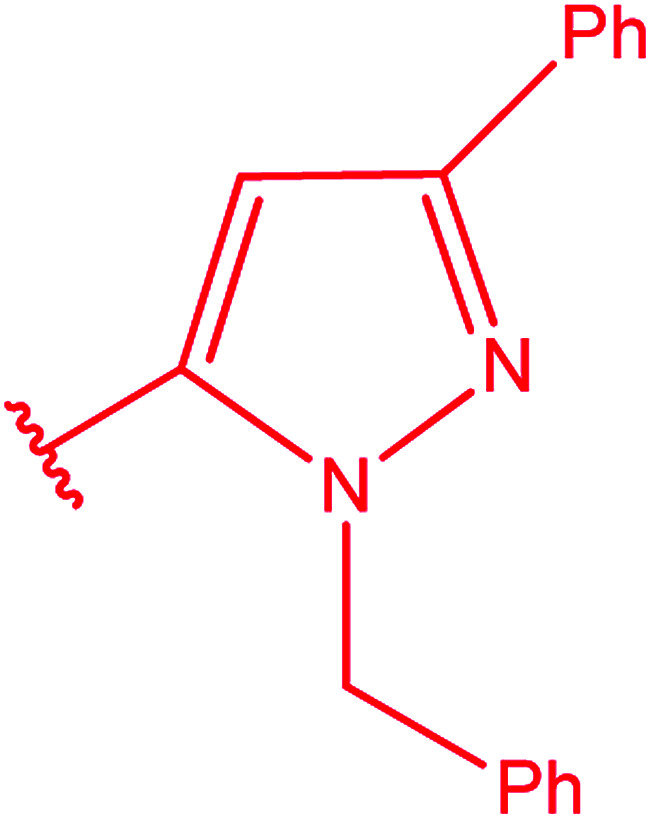	132
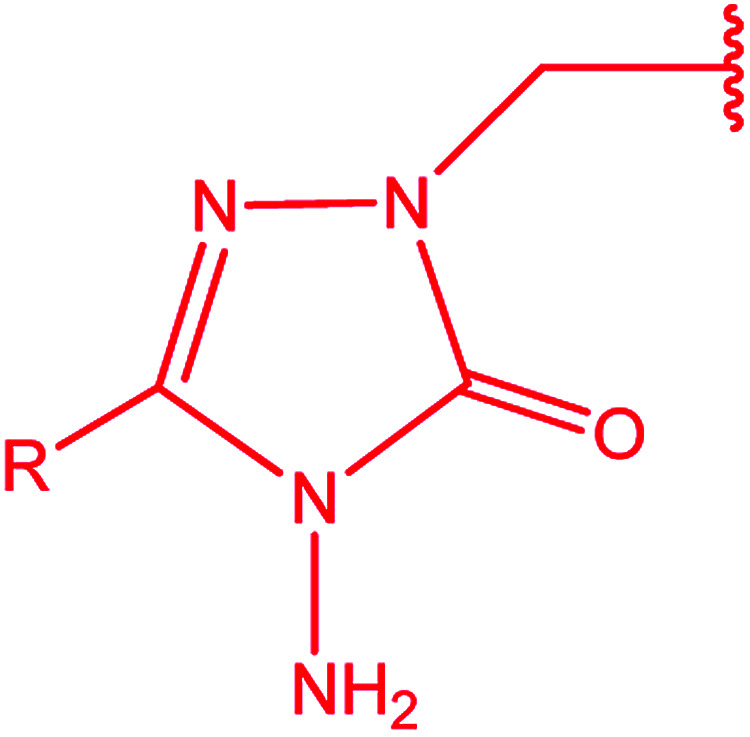	48
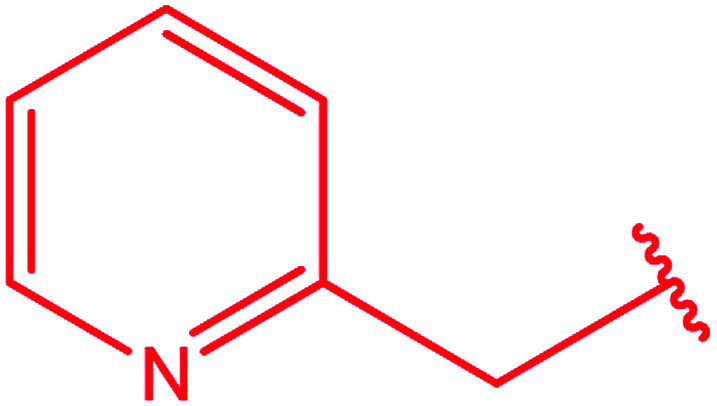	133
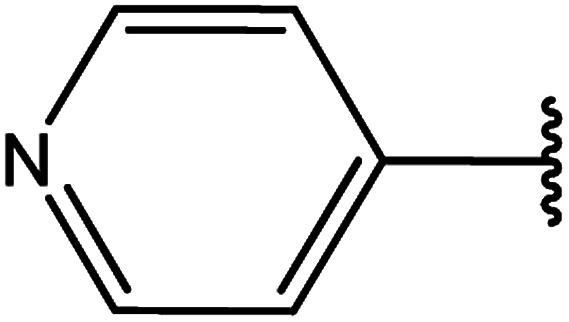	134
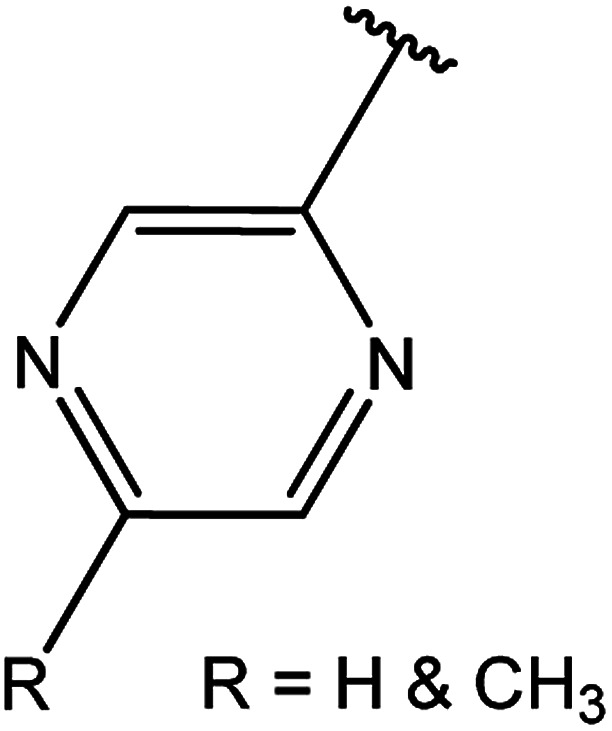	135
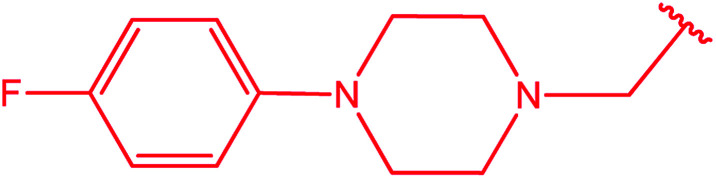	136
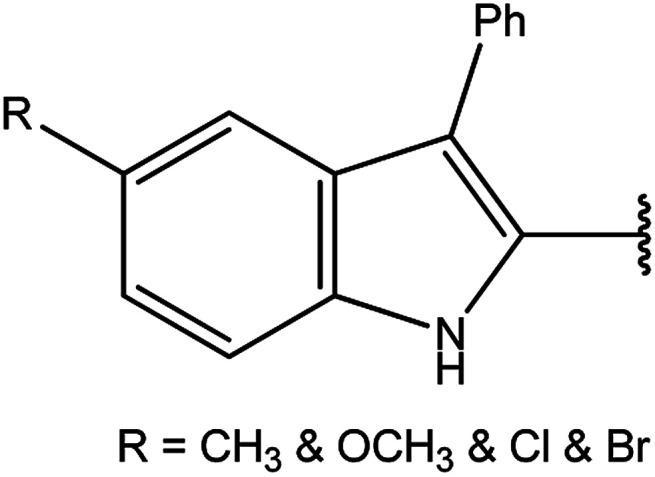	137
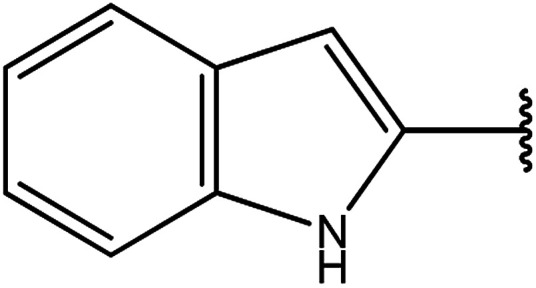	138
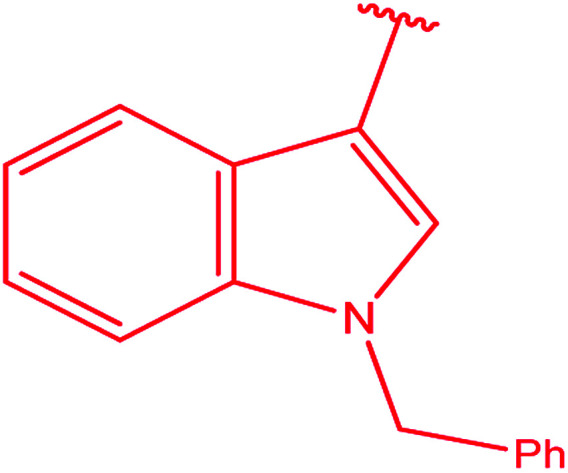	139
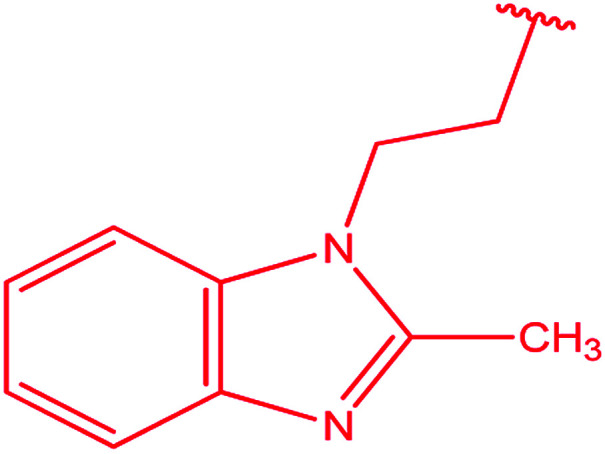	140
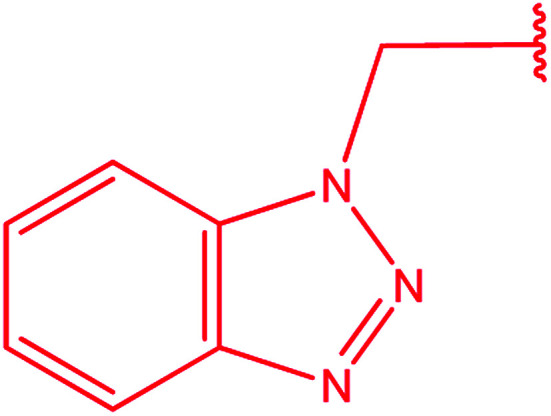	141

**Scheme 16:**
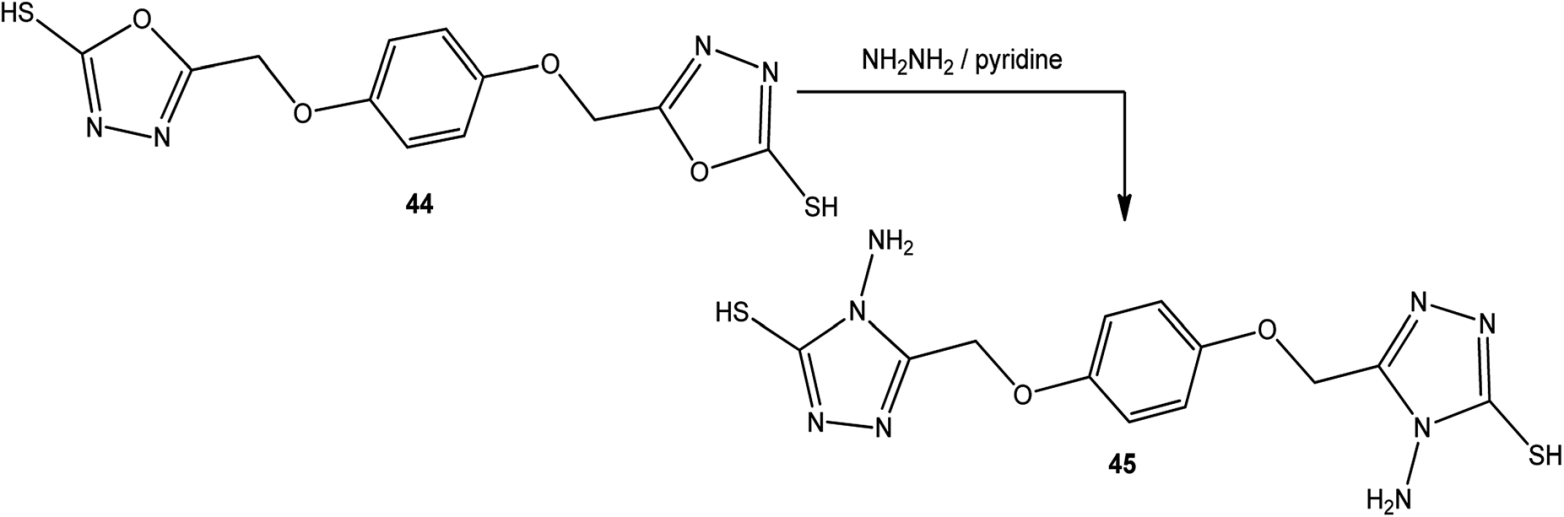
Synthesis of bis-triazole 45.

5,5′-[1,4-Phenylenebis(oxymethylene)]-bis(1,3,4-oxadiazole-2-thiol) (44) was converted into 5,5′-[(1,4-phenylenebis(oxymethylene)]-bis(4-amino-4*H*-1,2,4-triazole-3-thiol) (45) upon treatment with hydrazine hydrate in dry pyridine under thermal conditions (Scheme 16).^142^

**Scheme 17:**
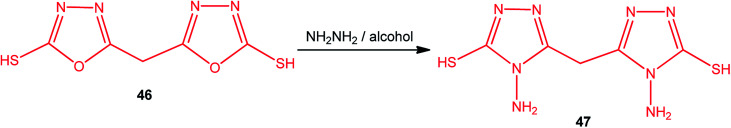
Synthesis of bis-triazole 47.

Similarly, the conversion of 5,5′-methylenebis(1,3,4-oxadiazole-2-thiol) (46) into 5,5′-methylenebis(4-amino-4*H*-1,2,4-triazole-3-thiol) (47) was achieved using an alcoholic hydrazine solution under refluxing conditions (Scheme 17).^1^

**Scheme 18:**
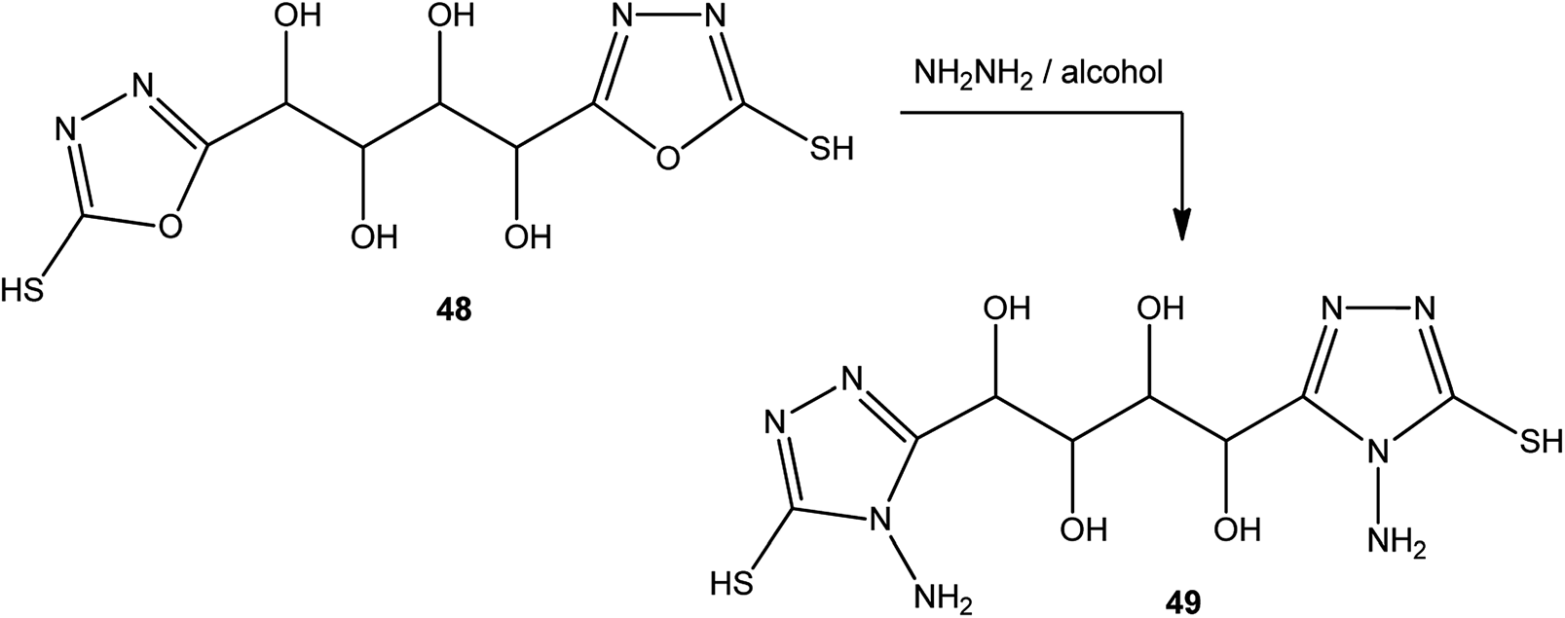
Synthesis of bis-triazole 49.

In addition, the same procedure (alcoholic hydrazine solution) was applied to the conversion of 1,4-bis(2-mercapto-1,3,4-oxadiazol-5-yl)butane-1,2,3,4-tetrol (48) to 1,4-bis(4-amino-5-mercapto-4*H*-1,2,4-triazol-3-yl)butane-1,2,3,4-tetrol (49) (Scheme 18).^143^

4-Amino-5-mercapto[1,2,4]triazole 1 and its 3-substituted derivatives 2–4 (Chart 2) contain both amino and mercapto groups as ready-made nucleophilic centers for the synthesis of condensed heterocyclic rings.

## 5. Conclusions and future directions

The reports in this review clearly demonstrate the elevated synthetic potential of 3-substituted-4-amino-5-mercapto[1,2,4]triazoles and bis-[4-amino-5-mercapto[1,2,4]triazoles]. Numerous scientific researchers in the fields of chemistry and pharmaceutical science are interested in the study and utilization of these compounds as building blocks in the synthesis of important bioactive compounds.

## Conflicts of interest

The authors declare that there is no conflict of interests regarding the publication of this paper.

## Supplementary Material
